# Exploring Binding Sites in Chagas Disease Protein TcP21 Using Integrated Mixed Solvent Molecular Dynamics Approaches

**DOI:** 10.1021/acs.jcim.4c01927

**Published:** 2024-12-17

**Authors:** William Oliveira Soté, Moacyr Comar Junior

**Affiliations:** Institute of Chemistry, Federal University of Uberlândia, Uberlândia 38400-902, Brazil

## Abstract

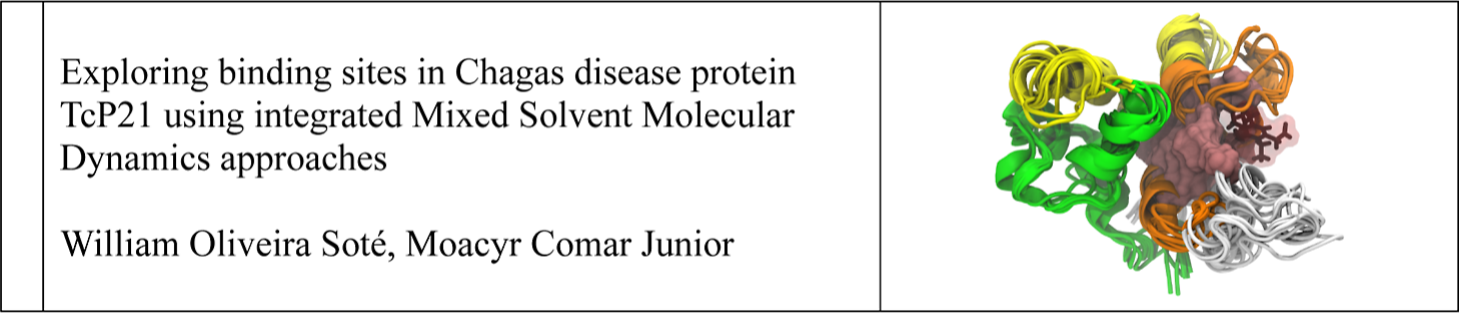

Chagas disease, caused by the protozoan Trypanosoma cruzi, remains a significant global health burden, particularly in Latin America, where millions are at risk. This disease predominantly affects socioeconomically vulnerable populations, aggravating economic inequality, marginalization, and low political visibility. Despite extensive research, effective treatments are still lacking, partly due to the complex biology of the parasite and its infection mechanisms. This study focuses on TcP21, a novel 21 kDa protein secreted by extracellular amastigotes, which has been implicated in *T. cruzi* infection via an alternative infective pathway. Although the potential of TcP21 for understanding Chagas disease is promising, further exploration is necessary, particularly in identifying potential binding sites on its surface. Computational tools offer a versatile and effective strategy for preliminary binding site assessment, facilitating a more cost-efficient allocation of experimental resources. In this study, we employed three independent computational approaches—mixed solvent molecular dynamics simulations (MSMD), fragment-based molecular docking, and pharmacophore model docking coupled with molecular dynamics simulations—to identify potential binding sites and provide comprehensive insights into TcP21. The three methodologies converged on a common site located on the external surface of the protein, characterized by key residues such as GLU55, ASP52, VAL70, ILE62, and TRP77. The protonated amino, acetamido, and phenyl groups of the pharmacophore probe were consistently observed to interact with the site via a network of salt bridges, hydrogen bonds, charge–charge interactions, and alkyl-π interactions, suggesting these groups play a significant role in ligand binding. This study does not aim to propose specific therapeutic hits but to highlight a still unknown and unexplored protein involved in *T. cruzi* cell invasion. In this regard, given the strong correlation between the three distinct approaches used for mapping, we consider this study offers valuable insights for further research into P21 and its role in Chagas disease.

## 1 Introduction

It has been 115 years since the discovery of the American trypanosomiasis, a tropical disease mostly known as Chagas disease, which is caused by the protozoan parasite Trypanosoma cruzi. A disease that not only predominantly affects socioeconomically vulnerable populations but also worsens economic inequality, contributes to their marginalization, and further reduces their already low political visibility.^1−3^ Despite this—or perhaps even because of it—Chagas disease has no cure or effective treatment. Although mainly found in Latin American countries, where an estimate of 6 to 8 million residents is infected, about 70 million people are at risk of infection, and causes 12 thousand annual deaths, it incurs around US$8 billion in annual economic loses, substantially impacting healthcare systems and compromising the productivity and working years of those infected.^4−6^ However, the burden of Chagas disease has extended beyond those primarily affected and has emerged as a global issue, requiring vigilance and care in many nonendemic countries.^7−11^ Efforts to combat Chagas disease and many other tropical diseases are now aligned with the global 2030 Agenda for Sustainable Development, particularly considering critical factors such as the steady increase in population migration and climate changes which are expected to lead to a considerable expansion of both infected people and the vectors of these diseases, respectively.^12,13^

The life cycle of the *T. cruzi* is well understood.^14^ In summary, it begins with the entry of the metacyclic trypomastigote form into the host’s bloodstream, via the feces of infected insect vectors (primarily triatomines) entering the bite site, or through the oral ingestion of the parasites.^15^ Once in the bloodstream, the metacyclic trypomastigotes invade host cells and transform into the amastigote form, establishing an infectious cycle. The amastigotes replicate, differentiate into bloodstream trypomastigotes, and are then released into the bloodstream, infecting additional cells. Each step in this biological cycle comprises numerous complex signaling pathways, with the infection mechanisms being dependent on the morphological form of the parasite, the wide range of strains, and the types of host cells to be invaded.^16−18^

Extensive research has demonstrated that, while each morphological form of trypomastigotes (metacyclic and bloodstream) utilizes distinct mechanisms for host-cell invasion, the associated signaling pathways consistently induce cytoplasmatic Ca^2+^ mobilization, which then triggers lysosomal exocytosis, and enables the parasite to exploit the release of intracellular contents for internalization into the cell.^19,20^ However, it has been observed that following cell invasion and subsequent lysis, the trypomastigote is not the only morphological expression secreted into the bloodstream; the amastigote is also present. Interestingly, this form also exhibits infective capacity rather than merely a replicative function, as previously assumed,^14,21,22^ which could prove critical for understanding the infection dynamics of the parasite and its persistence in infected organisms.^23−26^

Given this context, da Silva et al.^27^ identified a ubiquitous 21 kDa protein, referred to as P21 or TcP21, secreted by extracellular amastigotes with high propensity for membrane adhesion. This protein has demonstrated the ability to induce phagocytosis by directly interacting with the CXCR4 chemokine receptor, a transmembrane GPCR associated with cell internalization signaling.^28−30^ Additionally, P21 has shown inhibitory correlations to the intracellular replication of amastigotes;^31^ its presence greatly increases metacyclic trypomastigote and extracellular amastigote invasion of HeLa cells;^27^ and participates in confining the parasite within the infected cells under stress conditions, promoting dormancy and thereby improving parasite survivability.^32,33^ While the potential of P21 in combating and understanding Chagas disease,^34^ as well as its applications in other biological contexts,^35−37^ is noteworthy, many aspects remain to be explored, particularly its three-dimensional structure and the identification of possible binding sites on its surface. To date, the P21 structure has yet to be determined through either experimental or computational methods, and consequently, no binding sites have been proposed.

Experimental identification of binding sites, such as the Multiple Solvent Crystal Structures (MSCS),^38−42^ is notably a demanding, costly and complex process, as it requires satisfying challenging requisites, including the combination of the protein structural information and addressing its limited solubility in certain organic solvents.^43−47^ Within this context, computational approaches to preliminarily assess potential binding sites have become a common practice, which has proven to be considerably successful.^48,49^ A wide range of methods and tools for predicting allosteric and modulation sites, assessing conformational changes in proteins, sampling the energy landscapes of different protein–ligand complexes, and analyzing protein dynamics within the context of this research^50^ exists. Despite the different methodologies,^51−55^ there is no consensus on the most suitable computational approach for investigating binding sites, as these methods are frequently combined and continually updated.^49^

Two commonly used computational approaches that parallel the experimental MSCS are the Mixed Solvent Molecular Dynamics (MSMD)^56−60^ and the fragment-based molecular docking.^61−64^ The MSMD method samples molecular events of a protein within an organic solvent–water environment, but it uses classical Molecular Dynamics (MD) simulations. While the information provided by this approach comes from a statistical perspective, it benefits from being faster, more cost-efficient, and less labor-intensive than the experimental approach, enabling a more focused use of experimental resources.^65−67^ Furthermore, MSMD provides in-depth insights into the most frequent types of intermolecular interactions between protein and probe molecules.^68−70^ Alternatively, the fragment-based molecular docking method relies on a rigid-body and continuum solvation models to blindly sample numerous binding modes of organic molecules^71^ around the entire protein using molecular docking, which are then categorized based on empirical energy functions.^72^ This approach is faster than MSMD, enabling its application to a wider range of organic probes, although at the expense of not accounting for the dynamic elements of protein–ligand–solvent interactions.^71^

Given the novelty of the P21 protein and its potential role in an important but unexplored alternative pathway for *T. cruzi* infection, along with the versatility of computational approaches for mapping protein binding sites, the present study employed three independent methodologies to gain a deeper understanding of P21 and identify potential surface binding sites for future research. The methodologies used, as detailed in the Materials and Methods Section, include: (1) the MSMD based on MixMD^73^ methodology, (2) the fragment-based molecular docking using FTSite,^74^ and (3) combining elements of both. The latter approach consisted in generating a pharmacophore model based on the MSMD results, followed by blindly docking it onto the P21 protein to determine multiple binding modes, and concluding with MD simulations to assess the dynamics of the binding interactions. Six different organic probes (acetamide, acetonitrile, acetate ion, benzene, ethanol, and methylammonium ion) were used for the MSMD simulations. To our knowledge, this is the first instance of integrating these independent methodologies to scrutinize the surface of a protein and conduct a comprehensive computational study of the P21 protein.

## 2 Materials and Methods Section

### 2.1 MD Simulations Setup

Throughout this study, MD simulations were frequently employed to sample molecular conformations, and their trajectories were used to analyze and characterize potential allosteric binding sites. The following setup was applied for all the MD simulations conducted. All simulations were performed in triplicates. Specific details for each case are provided in their respective sections below.

The system was initially subjected to an energy minimization using the steepest descent algorithm, with a step size of 0.1 Å and a maximum force tolerance of 50.0 kJ mol^–1^ Å^–1^. After reaching a local minimum, a temperature annealing treatment was conducted in isochoric-isothermal (*NVT*) ensemble. It consisted of a linear temperature increase from 0 to 310 K over 400 ps, followed by 200 ps at 310 K, coupled using the velocity-rescaling method^75^ with a time constant for coupling of 0.1 ps. To gradually relax the protein structure within the chemical environment, this process was repeated three times. Initially, the motion of all nonhydrogen atoms in the protein was retrained with a force constant of 10 kJ mol^–1^ Å^–2^, progressively reducing it to 5 and then to 1 kJ mol^–1^ Å^–2^. Following the temperature treatment, a pressure of 1.0 bar was isotropically applied in isobaric–isothermal (*NPT*) ensemble for 1000 ps. The pressure was coupled using the Berendsen method,^76^ with a time constant for coupling of 2.0 ps and an isothermal compressibility of 4.5 × 10^–5^ bar^–1^. During this coupling, the restraining force constant remained at 1 kJ mol^–1^ Å^–2^. Once temperature and pressure had converged, all restraints were removed, and the system was sampled during 100 ns. Pressure coupling in this phase was achieved using the Parrinello–Rahman method.^77,78^ The short-range van der Waals interactions were limited to a cutoff radius of 11 Å. The long-range electrostatic interactions were evaluated using the smooth particle-mesh Ewald (SPME) algorithm^79,80^ and long-range dispersion corrections for the repulsive term of the Lennard–Jones equation were applied for energy and pressure. The bond stretching and bending motions were constrained using the parallel linear constraint solver (P-LINCS).^81^ Periodic boundary conditions were applied in all directions, and the classical equations of motion were integrated using the leapfrog algorithm. Data from the system was recorded every 2 ps. The energy minimization, equilibration, and sampling were conducted using GROMACS^82^ version 2019.2 and all-atom OPLS force field.^83^ The transferable intermolecular potential with 4 points (TIP4P)^84^ water model was used as solvent, and sodium and chloride ions were added to neutralize the charge of the system.

### 2.2 P21 Structure and Equilibration Setup

Since the P21 protein structure has not yet been determined experimentally, the Robetta Web server was used to estimate an initial conformation based on the publicly available FASTA sequence of the protein (GenBank ABS31351.1). The prediction was performed using the deep learning-based method RoseTTAFold.^85^ Five models were provided by the Robetta Web server, and the structure with the optimal structural error estimate and residue distribution in the Ramachandran plot was selected for equilibration. The structural comparison between the five models is available in the Supporting Information. The AlphaFold3^86^ method was also considered for this study; however, a comparative structural quality assessment of the models from RoseTTAFold and AlphaFold3 indicated that the AlphaFold3 model exhibited more structural uncertainties, particularly in the *N*-terminal region. In our interpretation, these uncertainties could hinder meaningful comparative analyses. Therefore, only the model from RoseTTAFold was selected for further experiments. Detailed information on the structural quality assessment is available in the Supporting Information. The selected model was then submitted to MD simulations to account for the effects of water, ions, temperature, and pressure on the predicted conformation. The initial conformation was centered within a 74.53 × 69.79 × 82.54 Å simulation box, ensuring a minimum distance of 15 Å between the protein and the box boundaries. A total of 41,732 particles was simulated. After the simulation, the sampled protein conformations were clustered using the single-linkage clustering method and the central structure was selected as the initial configuration for all subsequent experiments.

### 2.3 Binding Site Identification

**Figure 1:**
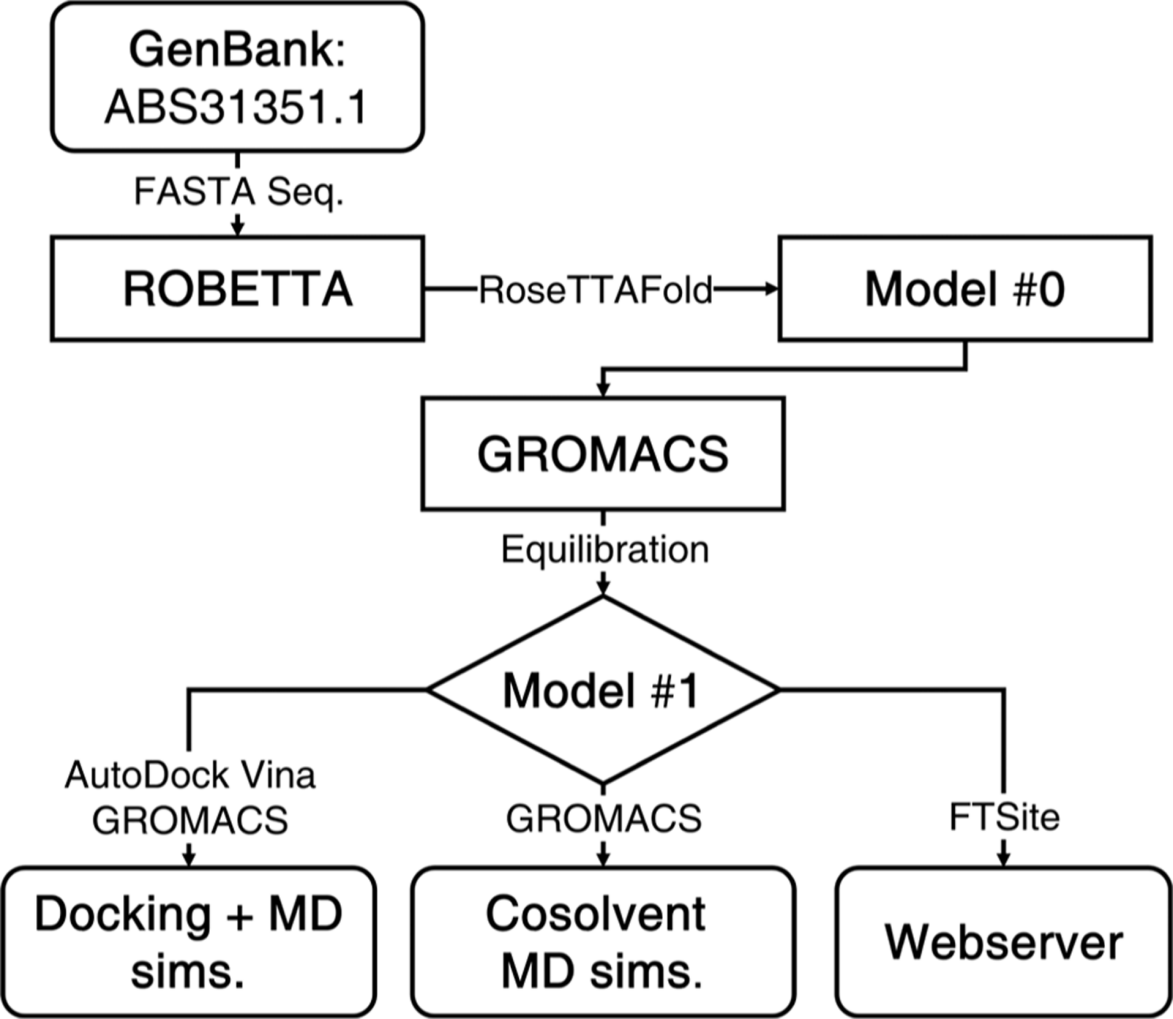
Flowchart of the experimental workflow.

To identify possible hotspots on the P21 protein surface, this study employed three independent approaches (Sections 2.3.1, 2.3.2, and 2.3.3). Our goal was to minimize bias inherent to any single methodology and mitigate the inaccuracies associated with the lack of experimental validation. By correlating them, we aimed to achieve more robust and reliable results. The general workflow used is illustrated in Figure 1, which includes both preceding and subsequent steps, as detailed below.

#### 2.3.1 MSMD Simulations

##### 2.3.1.1 Organic Solvent Structures and Force Field Description

Six organic solvents were selected for the MSMD simulations: acetamide (ACM), acetonitrile (ACN), acetate ion (ACT), benzene (BNZ), ethanol (ETH), and methylammonium ion (MAM), based on their miscibility in water, diverse range of intermolecular interactions (polar, nonpolar, and ionic), and frequent use in the reported literature.^57,87,88^ The initial conformation and the molecular mechanics parameters were determined using the automatic OPLS/CM1A parameter generator for organic ligand^89^ Web server. Each solvent was subjected to three iterations of conformational molecular optimization and the protonation states were modeled according to the physiological pH.

##### 2.3.1.2 System Configuration

**Figure 2:**
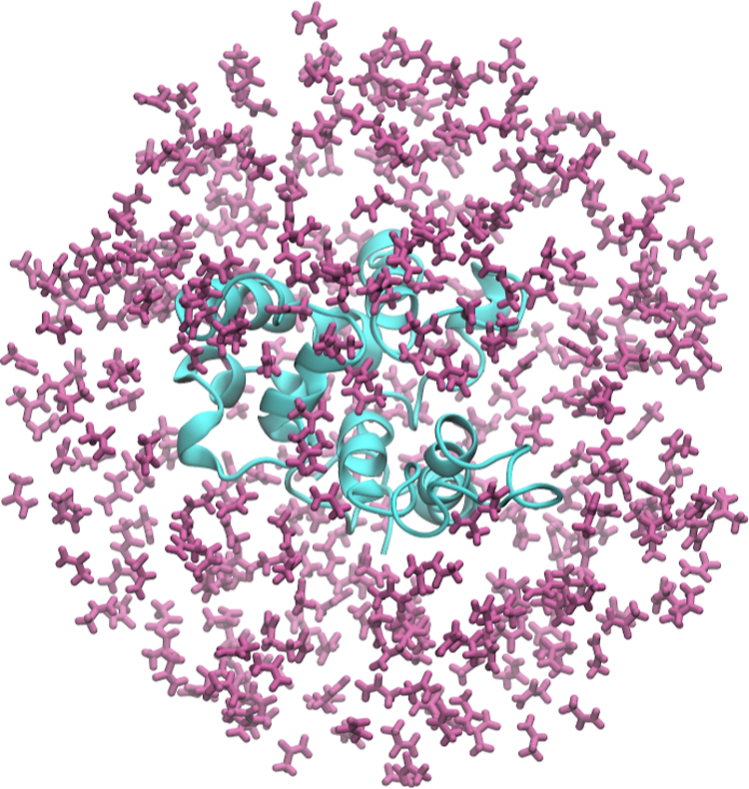
Schematic representation of the system configuration. The organic solvent is shown in mauve, the P21 protein is shown in cyan, and the water molecules are omitted for clarity.

The initial system configurations were built using PACKMOL.^90^ The selected concentration of organic solvent was 5% vol./vol., a value reported to increase hotspot detection over spurious site detection.^67,91^ The protein conformation obtained in Section 2.2. was placed at the center of a simulation box, and sufficient organic solvents were added to achieve the desired vol./vol. ratio. The molecules were randomly distributed within a spherical volume around the protein, as shown in Figure 2, to maximize the probability of interactions between the P21 and the organic solvents. The specific numbers of organic solvent and water molecules, as well as their molar mass, are shown in Table 1. The organic solvents were not mixed with each other.

**Table 1:** Number of Organic and Water Molecules in a 5% vol./vol. Concentration^a^

number of molecules		
organic solvent		water
acetamide (ACM)	371	20,034
acetonitrile (ACN)	364	20,020
acetate ion (ACT)	339	20,001
benzene (BNZ)	213	20,022
ethanol (ETH)	328	20,008
methylammonium ion (MAM)	393	20,043

a Water: 18.015 g mol^–1^; ACM: 59.068 g mol^–1^; ACN: 41.053 g mol^–1^; ACT: 59.044 g mol^–1^; BNZ: 78.114 g mol^–1^; ETH: 46.069 g mol^–1^; MAM: 32.066 g mol^–1^.

##### 2.3.1.3 MSMD Setup

Mixed solvent simulations were conducted in triplicates of 100 ns each, for every organic solvent–water mixture. A total of 18 unique systems were simulated, representing 1.8 μs of combined simulation time. The total number of simulated particles across all systems ranged from 62,528 to 66,143.

#### 2.3.2 Pharmacophore Model Docking

The organic solvents selected for the MSMD simulations were intended to serve as analogs for the pharmacophore features typically found in drug candidates; however, they do not account for key elements of drug-oriented interaction mapping, such as steric effects and the competition between multiple, diverse pharmacophore features within the same molecule. To address these limitations, a hypothetical pharmacophore test probe was created. Although the test probe does not represent an actual drug candidate, it enables investigating potential P21 interaction sites from an alternative perspective by mimicking a drug-like ligand. This novel and simplified modeling approach was inspired by more sophisticated methodologies commonly used in drug design, including those described by Giordano et al.^92^

##### 2.3.2.1 Probe Structure and Force Field Description

**Figure 3:**
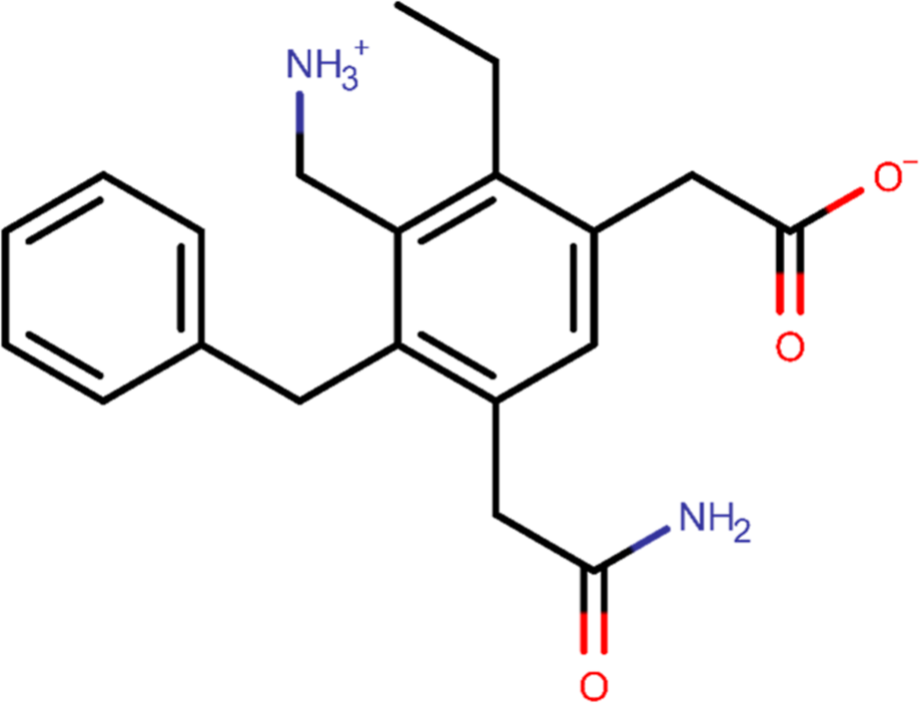
Pharmacophore test probe model.

The pharmacophore model, shown in Figure 3, was designed to accommodate functional groups analogous to those of the organic solvents previously used in the MSMD simulations, namely hydrophobic (aromatic and aliphatic), carboxylate, acetamido, and protonated amino groups. Protonation states were considered based on the physiological pH.

The molecular geometry was energy minimized using the theoretical model B3LYP^93^/6-31G^94^ using G09 software (Gaussian, Inc., Wallingford CT, 2016). The partial atomic charges were obtained based on the electrostatic potential-derived calculation scheme CHELPG.^95^ The automatic OPLS/CM1A parameter generator for organic ligands^89^ web server was employed to obtain the bonded and nonbonded molecular mechanical parameters, except for the partial atomic charges already calculated. Prior to docking the test probe onto the surface of the protein, it was thermodynamically equilibrated using MD simulations. The simulated system had a total of 12,157 particles. After equilibration, all sampled probe conformations were clustered to obtain a representative conformation. This conformation was subsequently used for the molecular docking.

##### 2.3.2.2 Molecular Docking

The molecular Docking was conducted using the AutoDock Vina^96,97^ software. To achieve blind docking, a search space of 120 × 100 × 126 Å was defined around the entire protein structure (from Section 2.2). An exhaustiveness level of 32 and a score function affinity energy threshold of 7 kcal mol^–1^ were chosen. Twenty unique runs were conducted, each with its own random seed. For each run, 20 binding modes were generated, resulting in a total of 400 binding modes.

##### 2.3.2.3 Molecular Dynamics Simulations Setup

The lowest energy binding mode was selected for further MD simulations, carried out in triplicates of 100 ns each, representing 300 ns of simulation time. The system had a total of 63,543 simulated particles.

#### 2.3.3 Webserver Prediction

To complement the prior experiments, an external approach was employed using the FTSite^71,74^ binding site predictor, with the equilibrated P21 conformation (Section 2.2) as the target. This Web server functions by docking 16 small organic molecular probes (ethane, ethanol, isopropyl alcohol, 2-methylpropan-2-ol, acetonitrile, methylamine, *N*,*N*-dimethylformamide, methyl ether, benzaldehyde, benzene, cyclohexane, phenol, acetamide, acetone, acetaldehyde, and urea) onto a rigid protein conformation, clustering the probes based on an energy function criterion, and identifying the predicted binding sites through the consensus clustering of the number of nonbonded interactions between the target and all probes in the cluster.

### 2.4 Analysis

The three sets of results (MSMD simulations, pharmacophore probe docking, and Web server prediction) were primarily analyzed using occupancy-based volumetric maps. Within the context of this study, spatial distribution functions offer a comprehensive overview of the most accessed regions on the protein surface by small and mobile molecules, such as the probes employed. Except for the Web server prediction, volumetric maps were calculated using VMD.^98^ The occupancies were calculated over the entire 100 ns of simulation, corresponding to 50,000 observed conformations. The fractional occupancies obtained after averaging across all frames were normalized using z-score calculations, as commonly employed in the literature,^69,87,91,99^ as follows
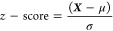
where ***X*** represents the individual fractional occupancies at each grid point, μ is the mean, and σ is the standard deviation across all grid points. Upon a visual evaluation of a range of occupancy thresholds to provide a clear representation of the volumetric maps for all ligands, a *z*-score of 20 was selected and is presented throughout the results.

The intermolecular interactions between the pharmacophore model and the P21 were categorized using Discovery Studio (BIOVIA, Dassault Systèmes, Discovery Studio, v.24, San Diego, 2023).

## 3 Results and Discussion

### 3.1 P21 Initial Conformation and Equilibration

The selection of the model for further experiments from the five generated by the Robetta Web server was based on two criteria: the residue structural error estimate profile provided by the Web server and the overall percentage distribution of residues in the Ramachandran plot, calculated using PROCHECK.^100^ The results, including the individual Ramachandran plots for each model, are presented in the Supporting Information.

**Figure 4:**
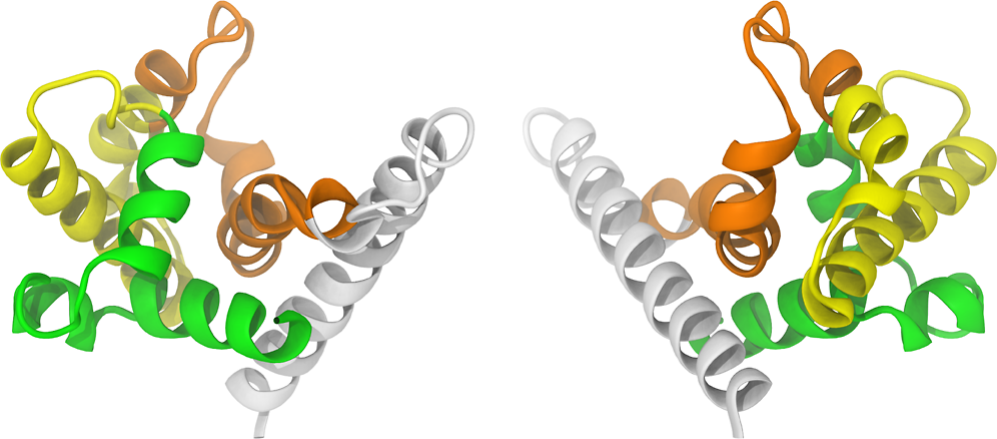
Cartoon representation of Model 2, one of the five structural models predicted by the RoseTTAFold algorithm. From left to right: front and rear views.

Given the lowest residue structural error estimate—particularly for residues 1 to 40 and 65 to 80—and the highest percentage of residues in the most favored regions (96.3%), the Model 2, shown in Figure 4, was selected as the initial conformation. The colors in Figure 4 were used solely for visual clarity and do not represent any specific characteristics. This color scheme will be consistently used to depict the P21 throughout the text.

**Figure 5:**
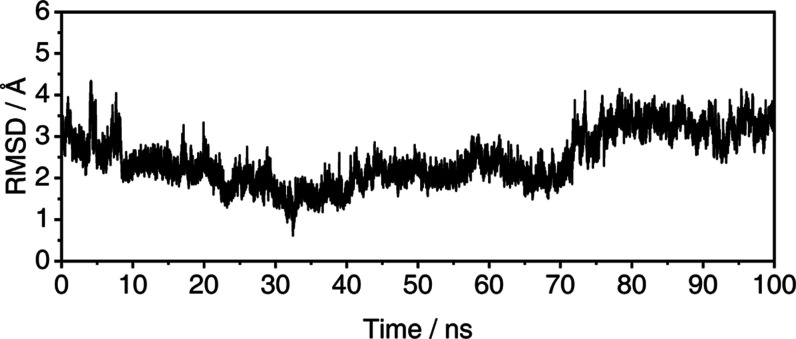
Average RMSD profile of the Model 2 backbone during the trajectory.

The predicted structure showed only two types of secondary structure: helices and turns. After the MD equilibration, the structural convergence of the Model 2 was first assessed through root-mean-square deviation (RMSD) calculation of its backbone, as depicted in Figure 5, which represents the average profile of the triplicates. Individual RMSD profiles are included in the Supporting Information (Figure S18).

**Figure 6:**
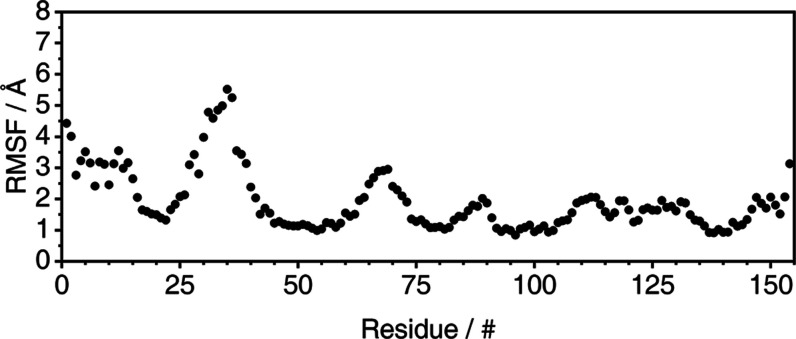
Average per residue RMSF profile of Model 2 backbone during the trajectory.

Figure 5 shows the initial conformational (at time = 0 ns) underwent substantial structural changes throughout the trajectory, indicating the chemical environment had a notable influence on the predicted structure. The mean ± standard deviation (SD) value was 2.46 ± 0.63 Å. The observed structural variations and the arguably high mean value were anticipated, given that the predictive algorithm does not account for chemical and thermodynamics factors, thereby providing an idealized conformation. To further investigate the structural profile of the P21 protein, root-mean-square fluctuation (RMSF) was calculated at a residue level, as shown in Figure 6, which also represents the average profile of the triplicates. Individual RMSF profiles are included in the Supporting Information (Figure S19).

Based on Figure 6, the P21 protein model exhibited groups of amino acid residues with considerably higher flexibility than others, particularly residues 1 to 15, 25 to 45, and 60 to 75. Specifically, residues 1 to 5, 25 to 44, and 65 to 73 are identified as turns. This increased flexibility is expected to result in higher RMSD values, as each data point of Figure 5 represents a global observation for the entire structure. Given this context, the RMSD profile may be interpreted as reasonably consistent throughout the trajectory, and both results can be correlated to support the argument for a relative structural convergence of the protein.

**Figure 7:**
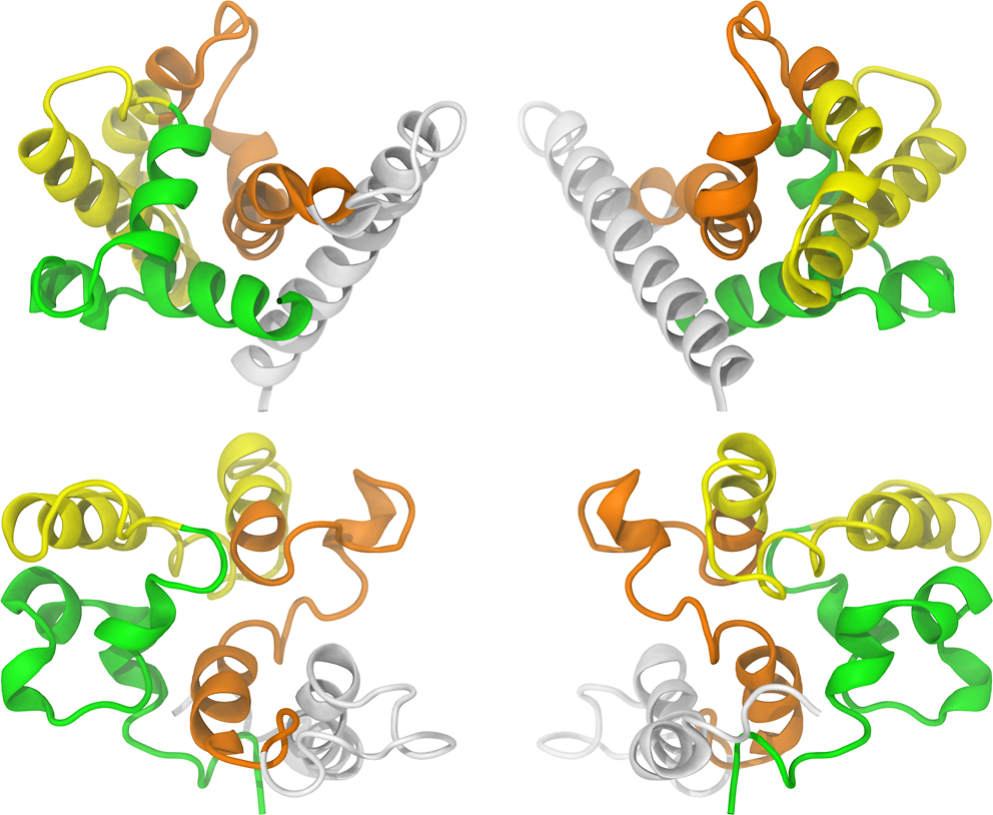
Cartoon representation of the P21 structure. From top to bottom: Model 2 and the clustered conformation. From left to right: front and rear views.

To obtain a representative conformation for further experiments, all sampled conformations from the trajectory were clustered. After the clustering calculation, the central structure, shown in Figure 7 alongside Model 2, was selected. Examination of this structure highlights the structural flexibility previously observed, as some of the initially predicted secondary structure is lost during the simulation. The observed structural difference can be attributed to the presence of the solvent molecules and their interactions with the protein. Nevertheless, the globular shape and the other conformational elements (e.g., certain helices) remained preserved through the simulated time frame.

### 3.2 Binding Site Identification

#### 3.2.1 MSMD Simulations

**Figure 8:**
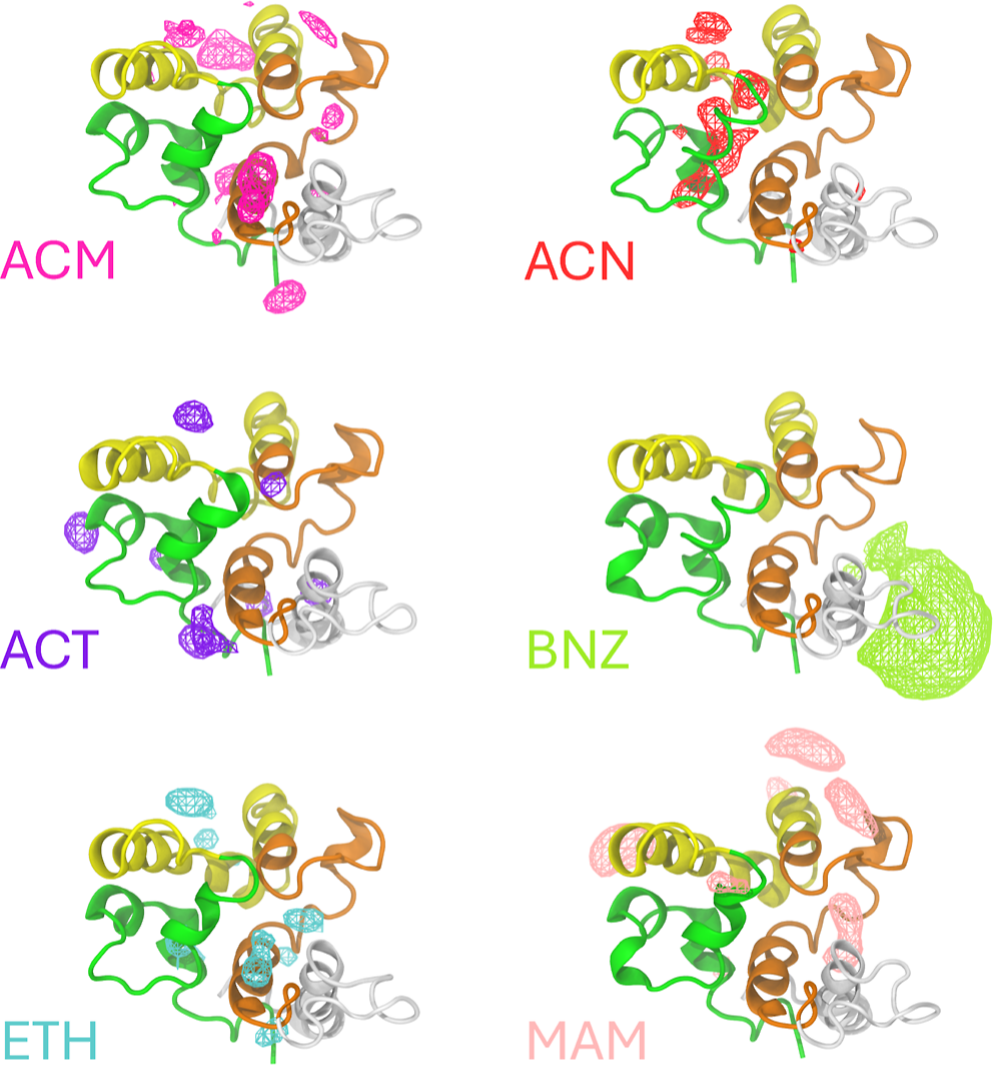
Occupancy-based volumetric maps of the respective organic solvents: acetamide (ACM), acetonitrile (ACN), acetate ion (ACT), benzene (BNZ), ethanol (ETH), and methylammonium ion (MAM).

Mixed solvent simulations were conducted to achieve an unbiased mapping of potential hotspots in the P21 structure. Binding site identification was carried out by calculating occupancy-based volumetric maps for each organic solvent and their respective replicas. The results are presented in Figure 8, shown as a function of the probe. For each system, the volumetric map represents the sum and intersection of the triplicates.

**Figure 9:**
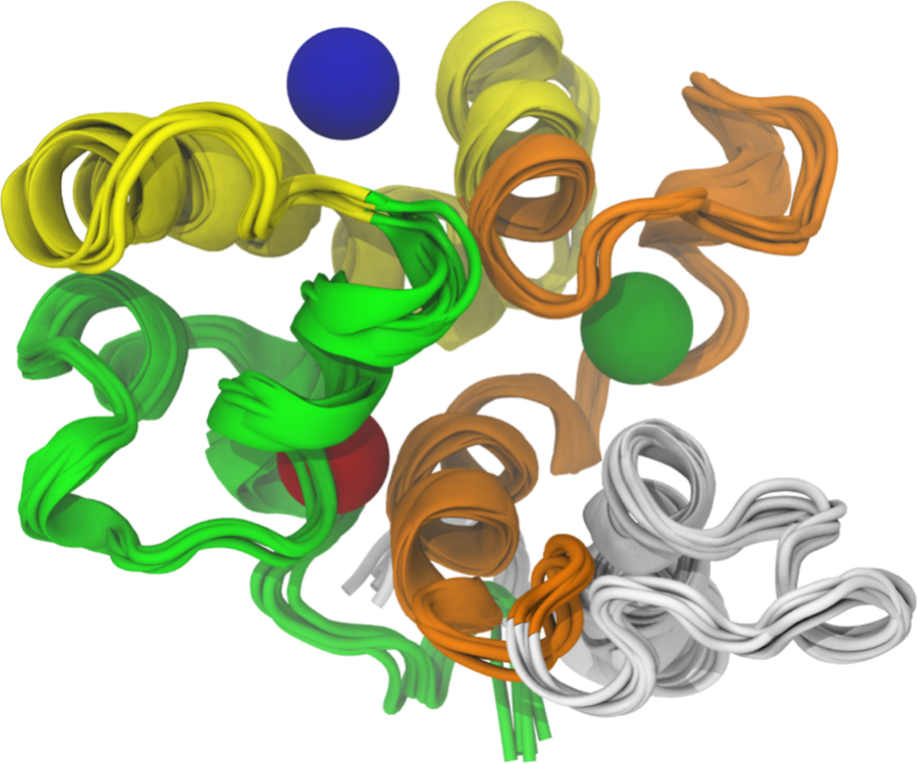
Cartoon representation of the superimposed P21 conformations from each solvent, with primary overlapping regions depicted as colored spheres.

Figure 8 illustrates that all probes occupied regions near the protein, both internally and externally, except for BNZ, which aggregated in a single external region around a hydrophobic moiety. Given the varying chemical properties of the probes, a global consensus was not anticipated; however, notable overlapping regions were observed. Three primary regions showing relative agreement were determined through qualitative assessment and are presented as colored spheres in Figure 9.

It is important to highlight the regions do not represent any specific order of probability but rather indicate overlap. The blue sphere overlapped with probes ACM, ACN, ACT, and ETH. Likewise, the red sphere overlapped with probes ACM, ACN, ACT, and ETH, although it appears slightly behind the green helix for probe ACT. Lastly, the green sphere overlapped with probes ACM, ETH, and MAM. Based on the organic probes, both regions exhibit polar, nonpolar, and ionic interactions. Given that P21 has not been explored prior to this study, there are no experimental observations to support the relevance of these sites. Therefore, it was imperative to corroborate these findings with those obtained using other methodologies. The individual RMSD profiles for each replica of the P21 protein in all simulated organic solvents are available in the Supporting Information (Figures S20–S25).

#### 3.2.2 Pharmacophore Probe Docking

**Figure 10:**
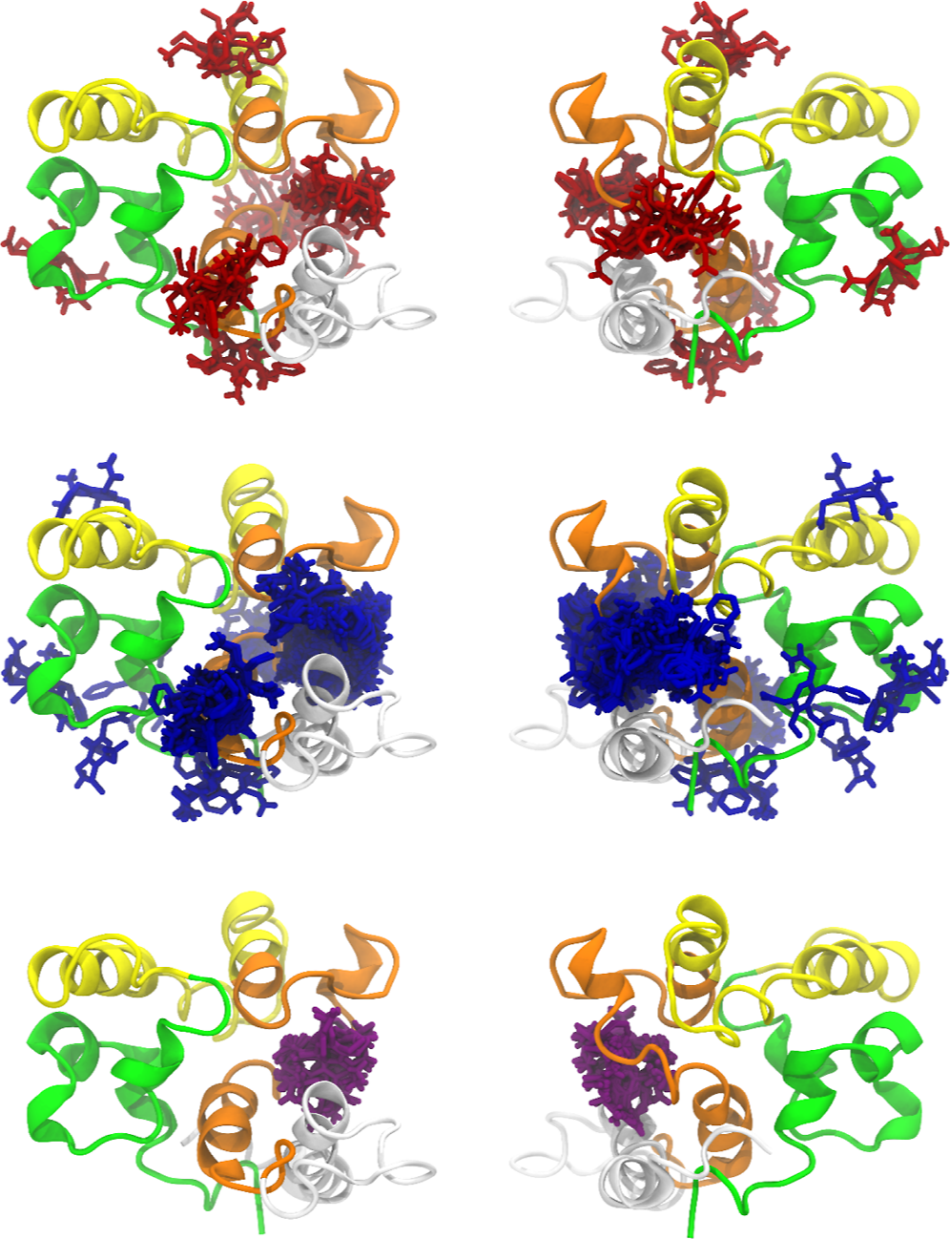
Schematic representations of binding modes organized by energy ranges. From top to bottom: > −5.0 kcal mol^–1^ between −5.0 and −5.9 kcal mol^–1^; and < −6.0 kcal mol^–1^. From left to right: front and rear views.

In the molecular docking experiment, out of the 400 generated binding modes, the energy range between the lowest and highest energy modes varied from −4.6 to −6.6 kcal mol^–1^. To elucidate the spatial distribution of binding modes around P21, the 400 generated conformations were organized by energy range and distinguished by color in Figure 10. Three groups were selected: (1) energies greater than −5.0 kcal mol^–1^, represented in red; (2) energies between −5.0 and −5.9 kcal mol^–1^, represented in blue; and (3) energies less than −5.9 kcal mol^–1^, represented in purple. The percentage energy distribution of the generated binding modes is shown in the Supporting Information (Figure S26).

According to Figure 10, the binding modes from groups (1) and (2) were scattered around different regions of the protein, with modes from group (2) being qualitatively more concentrated between the orange and white arrangements on the P21 structure. In contrast, as the affinity energy decreases, represented by group (3), all modes were confined within a specific pocket. This pocket is not exclusive to group (3) but exhibited considerably less variance compared to the other groups. Given this distinct distribution, only the lowest affinity energy was considered for further simulations.

**Figure 11:**
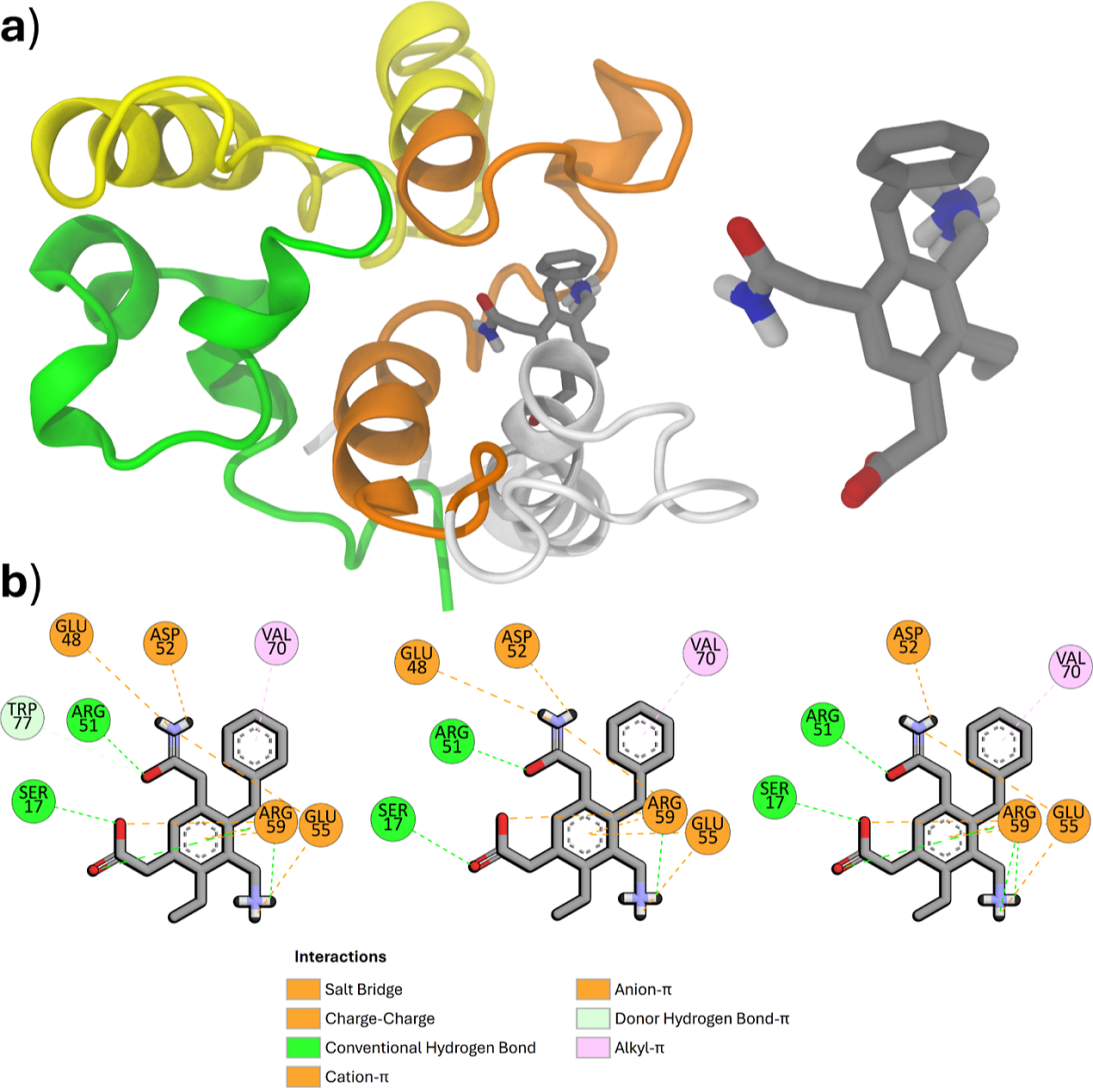
(a) Lowest energy binding modes with detailed probes, and (b) the 2D diagrams of protein-probe interactions.

The lowest affinity energy was −6.6 kcal mol^–1^, observed in three binding modes from distinct runs. Their conformations, as shown in Figure 11a, were similar and arguably interchangeable, with a maximum RMSD value of 0.57 Å. The main structural difference was in the rotation angle of the amino group. Notably, these binding modes were situated near the region of the green sphere identified previously. Their intermolecular interactions were also similar, as shown in Figure 11b, classified using Discovery Studio. The primary types of intermolecular interactions identified were electrostatic (including salt bridge, charge–charge, cation-π, and anion-π interactions) and hydrogen bonding (both conventional and salt bridge types). The only differences between the modes were the presence of residue TRP77 (donor-π interaction) in only one, and residue GLU48 (charge–charge interaction) in two. Overall, the binding modes were considered equivalent, and the selection for further simulations was made arbitrarily.

**Figure 12:**
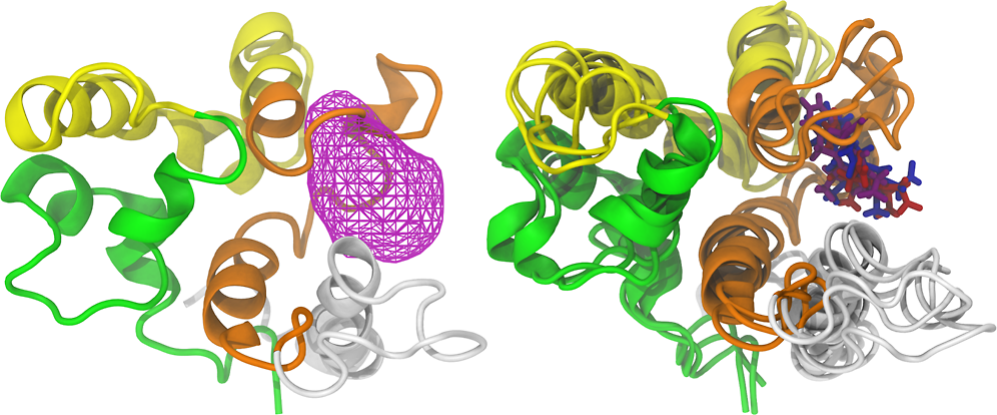
From left to right: occupancy-based volumetric map and superimposition of the clustered conformations.

After MD simulations, the interaction between the pharmacophore ligand and the binding site was also assessed using occupancy-based volumetric maps. The results, presented in Figure 12, include the volumetric map representing the sum and intersection of the triplicates, along with the superimposed clustered central structures from each replicate.

**Figure 13:**
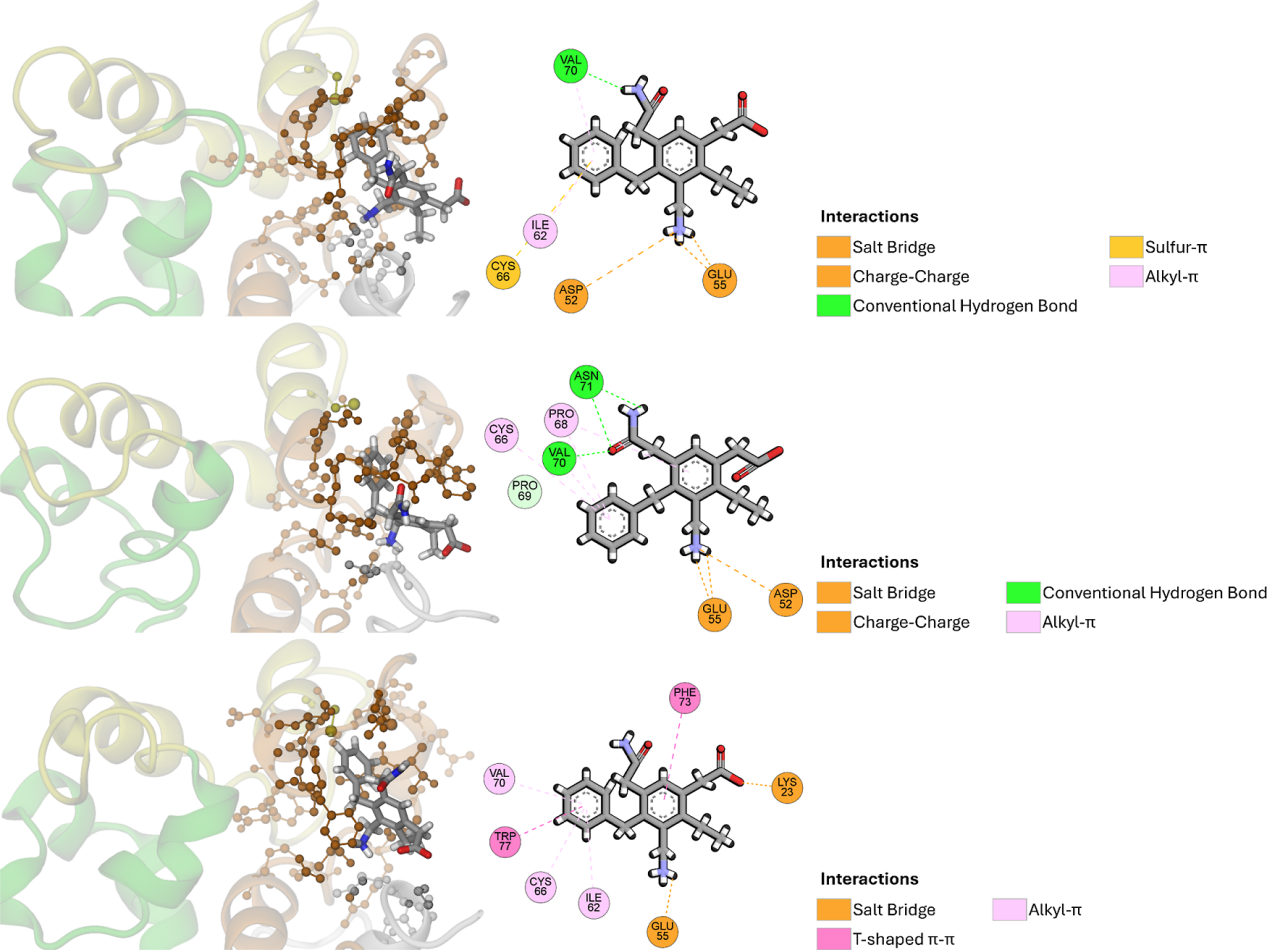
Clustered central structures from each replica and their corresponding 2D diagrams of protein-probe interactions.

In contrast to the volumetric maps of the MSMD simulations, which assessed the occupancy of multiple molecules, Figure 12 is specific to a single molecule, thus exhibiting a clearer spatial distribution. This occupancy profile suggests that the pharmacophore model remained relatively close to its initial site over the sampled time range, likely due to significant interactions, which will be discussed in detail later. A minor shifting of the interaction region was anticipated, given that the molecular docking did not account for external influences such as solvent, temperature, pressure, and ions, nor was the protein treated as a flexible body. Nonetheless, the identified region still closely matches the green region observed in the MSMD simulations (Figure 9). For the specific interactions between the probe and P21, 2D diagrams were created for the central clustered conformation of each replica using Discovery Studio. They are shown in Figure 13, directly next to their respective conformations.

In contrast to the interaction diagrams preceding the MD simulations, the replicas this time exhibited notably different intermolecular interactions among themselves, despite the pharmacophore model binding modes being relatively close to each other in relation to P21. The residues GLU55, CYS66, and VAL70 were consistently present across all replicas, suggesting their significant role in maintaining probe stability and identifying them as potential hot spots. Particularly, the residue GLU55 presented charge–charge and salt bridge interactions with the protonated amino group in all cases, and residue CYS66 interacted with the phenyl group through sulfur-π interactions in the first replicate, and alkyl-π interactions in the latter two. Similarly, residue VAL70 interacted with the phenyl group through alkyl-π interactions in both replicas, in addition to hydrogen bonding in two replicas: first as an acceptor for the amine from the acetamido group, and second as a donor for the carbonyl from the same group. It is important to highlight that when residue VAL70 acted as a donor for the acetamido group, residue ASN71 also interacted with the same group, serving both as an acceptor and donor in hydrogen bonding, thereby creating an interactive network among them. Besides these three residues, ASP52 and ILE62 were observed in two of the three replicates. ASP52 predominantly engaged in charge–charge interactions with the protonated amino group, whereas ILE62 through alkyl-π interactions with the phenyl group. The other observed interactive residues were specific to each replica. Both pre and postMD replicas consistently highlighted the potential importance of the moiety consisting of the protonated amino, acetamido, and phenyl groups, which acted as a fixation point within the binding site for the pharmacophore probe. Meanwhile, the carboxylate and the aliphatic groups remained oriented away from the binding site. The individual RMSD profiles for each replica of the P21 protein with the pharmacophore probe are available in the Supporting Information (Figure S27).

**Figure 14:**
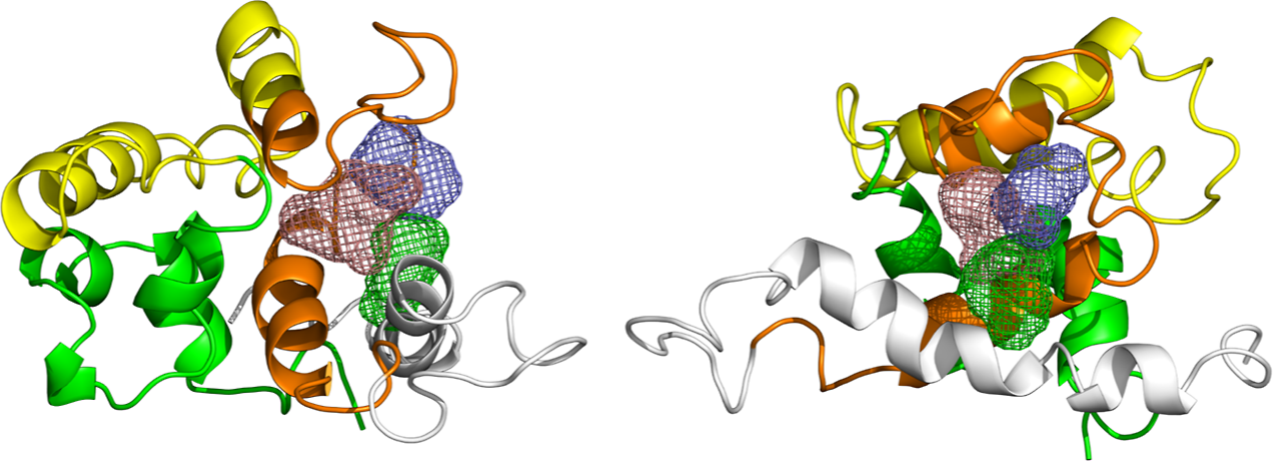
Predicted binding sites using the FTSite Web server. From left to right: front and side views.

To further investigate the possibility of additional binding sites on the P21 structure and to complement the MSMD and pharmacophore probe simulations, a third predictive tool, the FTSite Web server, was employed. The results are presented in Figure 14.

**Figure 15:**
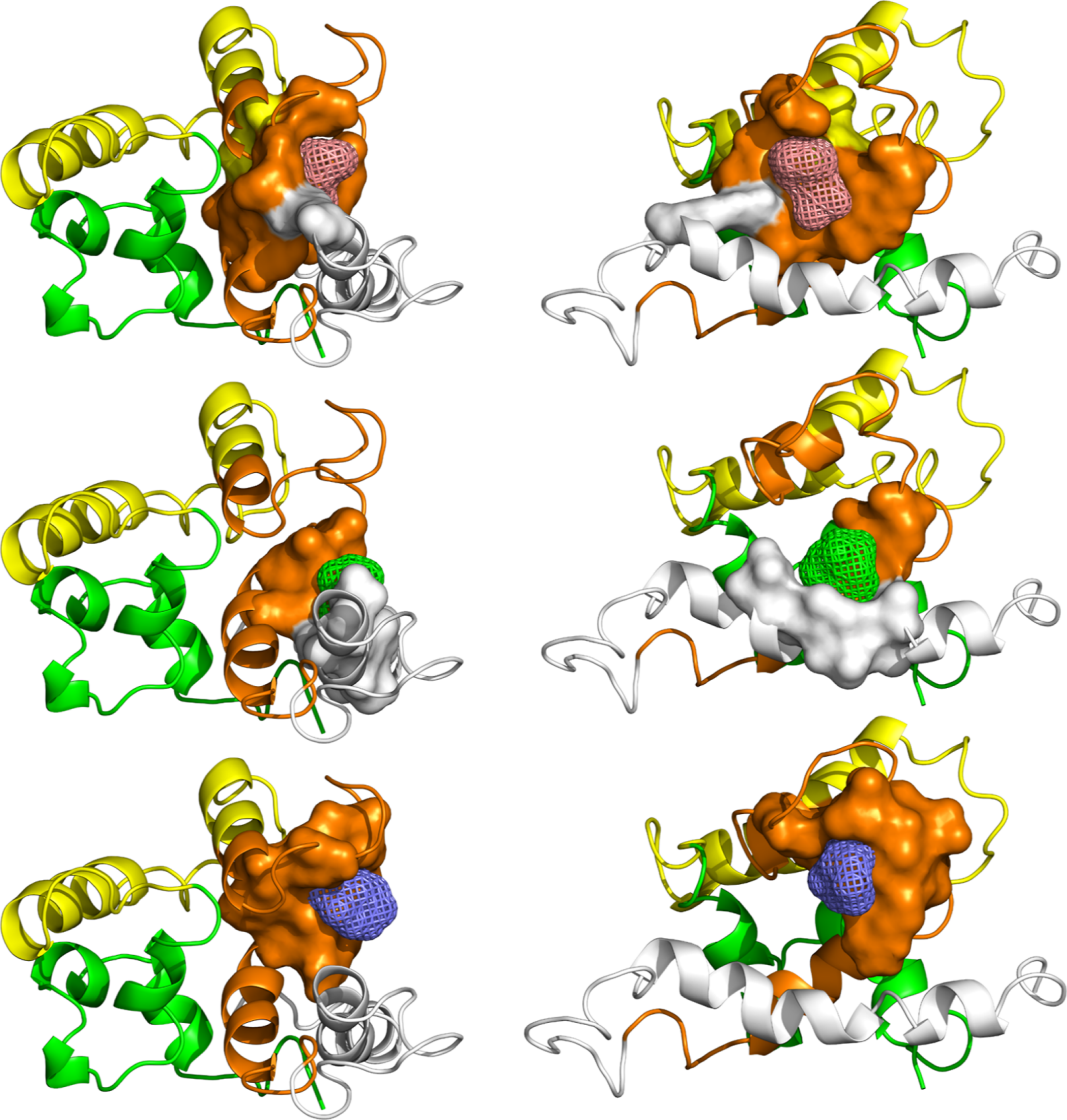
Predicted binding sites using the FTSite Web server, with interactive protein residues and consensus clusters shown as solid and mesh surfaces, respectively. From left to right: front and side views.

Three potential binding sites were found and are depicted in Figure 14. Each site corresponds to a consensus cluster, ranked by the number of nonbonded interactions between the target protein and the 16 organic probes used by the FTSite Web server. The clusters are colored salmon, green, and blue, representing consensus values of 93.7%, 87.5%, and 62.5%, respectively. It is important to notice the predicted binding sites were located near those identified by the previous methods (Figures 9–12), supporting their importance. The predicted binding sites were further detailed in Figure 15, where the interacting protein residues were qualitatively shown as individual surface representations.

A qualitatively assessment of Figure 15 indicates that each cluster primarily interacts with distinct portions of the pocket located between the orange and white colored regions of the P21 protein, while still sharing some residues, particularly in the orange region. Based on the figure, the salmon cluster appears to access a deeper pocket than the other clusters, which would explain the observed differences in consensus values, as a deeper pocket would likely mean interacting with more residues. To complement this assessment, the interacting protein residues for each cluster were listed in Table 2, with common residues (present in multiple clusters) and unique residues (those present in only one cluster) highlighted.

**Table 2:** Interacting Common and Unique Residues from P21 and Their Corresponding Regions

cluster 1 (salmon)	cluster 2 (green)	cluster 3 (blue)
*common residues*		
ASP52	ASP52	
GLU55	GLU55	GLU55
ARG59	ARG59	ARG59
VAL70		VAL70
TRP77		TRP77
*unique residues*		
	ALA13	
	CSY14	
	SER15	
	VAL16	
	SER17	
	GLU20	
ARG24		
GLU48		
ARG51		
THR54		
		CYS58
		GLU60
		GLU61
		ILE62
		THR63
PHE73		

Table 2 corroborates the observation that although most interactive residues were found in distinct portions of the pocket, a few residues are shared between the clusters. Notably, residues GLU55 and ARG59 appeared in all FTSite clusters, while ASP52, VAL70, and TRP77 appeared in two. A total of 16 different residues were involved in unique interactions and at least 5 were common among two or more clusters. The salmon cluster, which showed the highest consensus value (93.7%), also showed the highest number of interactive residues, with 10, whereas the other clusters showed 9.

In comparison to the previous results, now summarized in Table 3, GLU55 was a ubiquitous in both pharmacophore probe docking and MD simulations, as well as in the FTSite Web server prediction. ASP52 and VAL70 were also common residues, with ASP52 appearing in two MD clusters and in two FTSite clusters, and VAL70 appearing in all MD clusters, two FTSite clusters, and most molecular dockings. ARG59 only appeared in molecular docking experiments. Interestingly, TRP77 appeared in one MD simulation cluster and in two FTSite clusters, and ILE62 appeared in two MD simulation cluster and in only one FTSite cluster.

**Table 3:** Summary of Common Interaction Residues from P21 Across the Used Methodologies^a^

molecular docking (pharmacophore probe)			MD simulations (pharmacophore probe)			molecular docking (FTSite)		
**BM1**	**BM2**	**BM3**	**R1**	**R2**	**R3**	**C1**	**C2**	**C3**
*common residues*								
SER17	SER17	SER17					SER17	
GLU48	GLU48					GLU48		
ARG51	ARG51	ARG51				ARG51		
ASP52	ASP52	ASP52	ASP52	ASP52		ASP52	ASP52	
GLU55	GLU55	GLU55	GLU55	GLU55	GLU55	GLU55	GLU55	GLU55
ARG59	ARG59	ARG59				ARG59	ARG59	ARG59
			ILE62		ILE62			ILE62
			CYS66	CYS66	CYS66			
VAL70	VAL70	VAL70	VAL70	VAL70	VAL70	VAL70		VAL70
					PHE73	PHE73		
TRP77					TRP77	TRP77		TRP77

a *Note*. The letter codes BM, R, and C represent “Binding Mode”, “Replica”, and “Cluster”, respectively.

**Figure 16:**
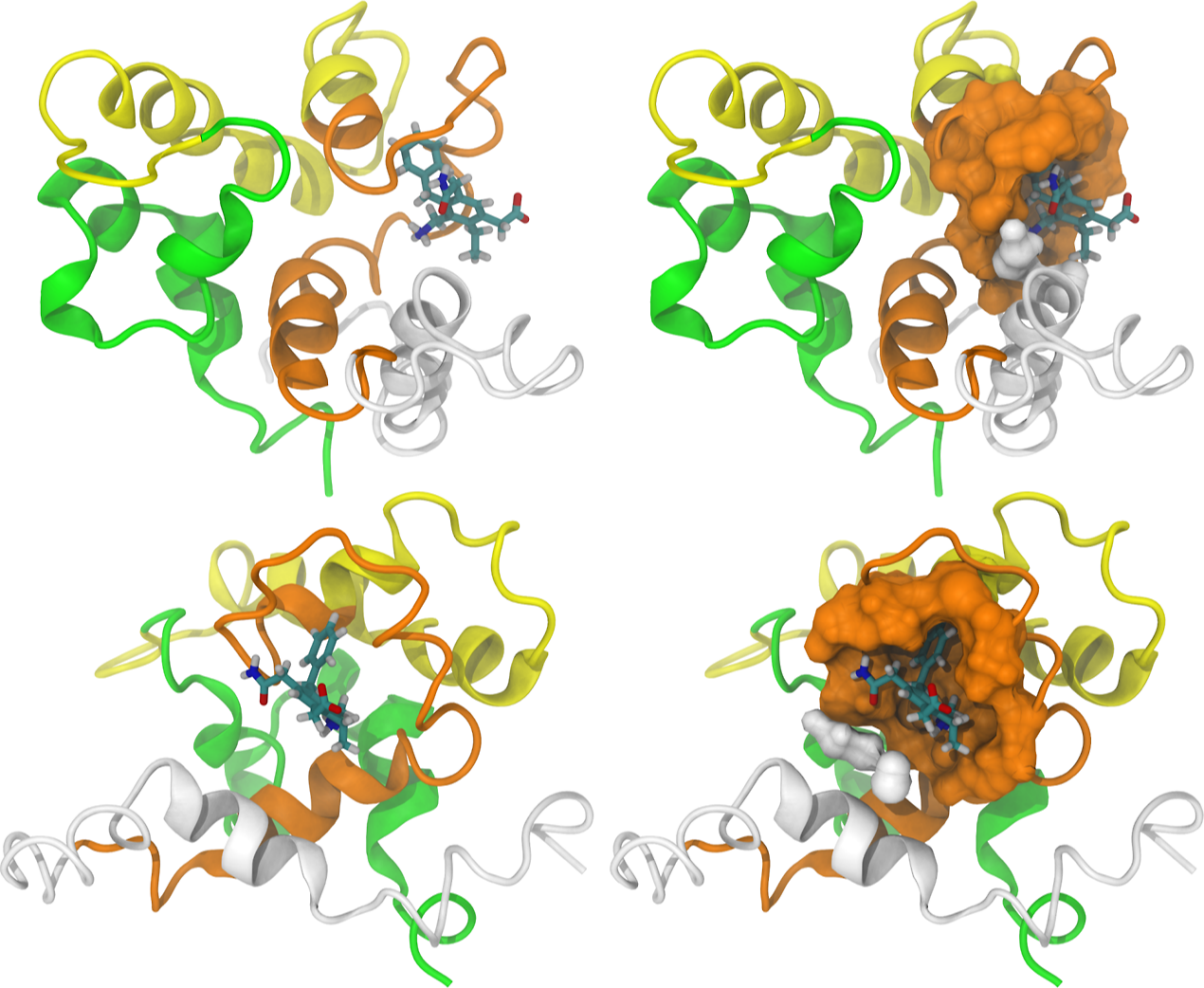
Qualitative binding site model for the pharmacophore probe. From top to bottom: front and side views. From left to right: cartoon and surface representations.

Based on the correspondence among MSMD simulations, the pharmacophore probe docking with MD simulations, and the FTSite Web server predictions, a qualitative binding site model was created, as shown in Figure 16. The reference system for perspective purposes was the cluster from an arbitrary replica of the MD simulations with the pharmacophore probe.

This model integrates all common residues previously observed across the replicas, and those within 5 Å of an arbitrary lowest energy pharmacophore model binding mode, used for perspective purposes. Located on an external surface of P21, this binding site seems to have no substantial steric hindrance that would obstruct the approach of a similar ligand. Finally, although this study was built on a robust technical foundation—integrating methodologies from several already cited studies on in silico binding site prediction—it is important to acknowledge its limitations. The absence of an experimentally solved three-dimensional structure for the P21 protein and data on its actual binding sites prevent complete validation of the proposed binding site and determination of the type of allosteric modulation involved (activation or inhibition), if present. However, despite these limitations, we believe our findings contribute valuable atomistic insights into this novel protein and highlight the significance of its role in Chagas disease.

## 4 Conclusions

Chagas disease remains a longstanding burden that profoundly impacts socioeconomic conditions of thousands of individuals. Despite ongoing research efforts, an effective treatment has yet to be identified. The development of such a treatment is hindered by many obstacles, such as the complex biological nature of the*T. cruzi* parasite and its infection mechanisms; the challenges of identifying novel protein targets involved in host-cell infection; and the discovery of potential binding sites that could be targeted to halt or mitigate the infection. Computational approaches for preliminary assessment of potential binding sites have demonstrated versatility, robustness, and effectiveness as a strategy for investigating proteins, thereby enabling a more targeted and efficient use of experimental resources.

In this study, three distinct computational approaches—mixed solvent molecular dynamics simulations, fragment-based molecular docking Web server prediction, and pharmacophore model docking coupled with molecular dynamics simulations—were employed to explore binding sites and provide in-depth insights into the novel P21 protein, within the context of Chagas disease. All three approaches identified a common binding site, featuring a hotspot with residues GLU55 and ASP52 (hydrophilic acidic), VAL70 and ILE62 (hydrophobic aliphatic), and TRP77 (hydrophobic aromatic). The binding site, located on the external surface, consistently interacted with the protonated amino, acetamido, and phenyl groups of the pharmacophore model. The observed interactions—salt bridges, hydrogen bonds, charge–charge interactions, and alkyl-π interactions—established an interactive network with the probe, suggesting an important role for ligand binding. In the absence of experimental validation of P21 binding sites, the findings presented should be regarded as preliminary observations, offering a novel perspective. Finally, we would like to emphasize this study does not aim to propose specific therapeutic hits but to highlight a still unknown and unexplored protein involved in*T. cruzi* cell invasion. In this regard, given the strong correlation between the three distinct approaches used for mapping, we consider this study offers valuable insights for further research into P21 and its role in Chagas disease.

## Data Availability

Data and Software Availability Content: The P21 FASTA sequence was obtained from the GenBank sequence database under accession number ABS31351.1 (https://www.ncbi.nlm.nih.gov/protein/ABS31351.1). The initial predicted conformation of the P21 was obtained from the Robetta structure prediction service (https://robetta.bakerlab.org). The conformations and molecular mechanics parameters for the organic solvents and pharmacophore model were obtained using the LigParGen web server (http://zarbi.chem.yale.edu/ligpargen). The optimized starting geometry and partial atomic charges of the pharmacophore model were calculated using Gaussian 09 (https://gaussian.com). Initial configurations for the MSMD systems were modeled using PACKMOL (https://m3g.github.io/packmol). All MD simulations were conducted with GROMACS (https://www.gromacs.org). Molecular docking studies were performed using AutoDock Vina (https://vina.scripps.edu). The Web server-based binding site prediction was performed by FTSite (https://ftsite.bu.edu). Data analysis was carried out using GROMACS, VMD (https://www.ks.uiuc.edu/Research/vmd), and BIOVIA Discovery Studio (https://www.3ds.com/products/biovia/discovery-studio). All simulation input files (.pdb, .itp, .top, and .mdp) are available at GitHub (https://github.com/mollab/p21-binding-site).

## Supporting Information Available

Structural quality assessment study; Residue Structural Error Estimate profile and percentage distribution residues in the Ramachandran plot for the models generated by the Robetta service; individual Ramachandran plot for the models; score function affinity energy-based percentage distribution of the binding modes calculated by AutoDock Vina (PDF)
