# Supplementary material for: Exploring Binding Sites in Chagas Disease Protein TcP21 Using Integrated Mixed Solvent Molecular Dynamics Approaches
Source: J Chem Inf Model. 2024 Dec 17;65(1):363–77. doi: 10.1021/acs.jcim.4c01927 (PMC11733930; doi:10.1021/acs.jcim.4c01927)
Supplement: Supplementary file 1 — ci4c01927_si_001.pdf [file ci4c01927_si_001.pdf]

SUPPORTING INFORMATION

FOR

Exploring Binding Sites in Chagas disease protein TcP21 using integrated Mixed Solvent Molecular Dynamics Approaches

William Oliveira Soté [a], Moacyr Comar Junior [a*]

[a] *Institute of Chemistry, Federal University of Uberlândia, Uberlândia, Brazil*

[*] Corresponding Author Email: mcomjr@ufu.br

1. STRUCTURAL QUALITY ASSESSMENT

1.1. Comparison between Models 1–5 from RoseTTAFold.

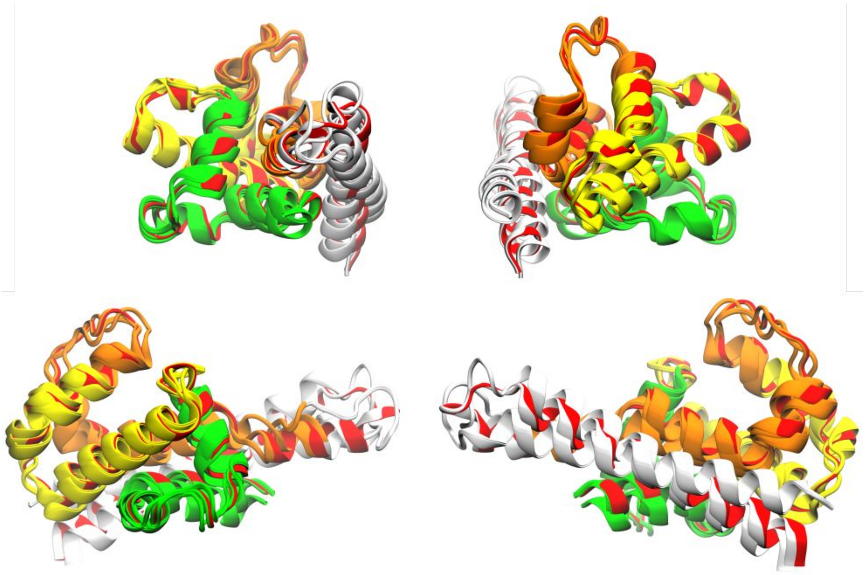

Figure S1. Structural alignment of the entire protein from Models 1–5 in different angles. Model 2 is highlighted in red for reference purposes.

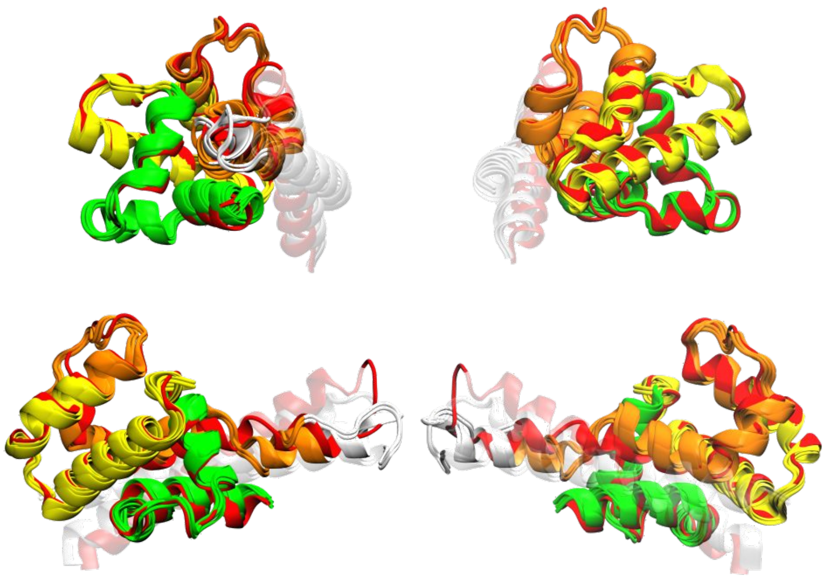

Figure S2. Structural alignment of residues 58–154 from Models 1–5 in different angles. Model 2 is highlighted in red for reference purposes. Residues 1–57 are shown transparent.

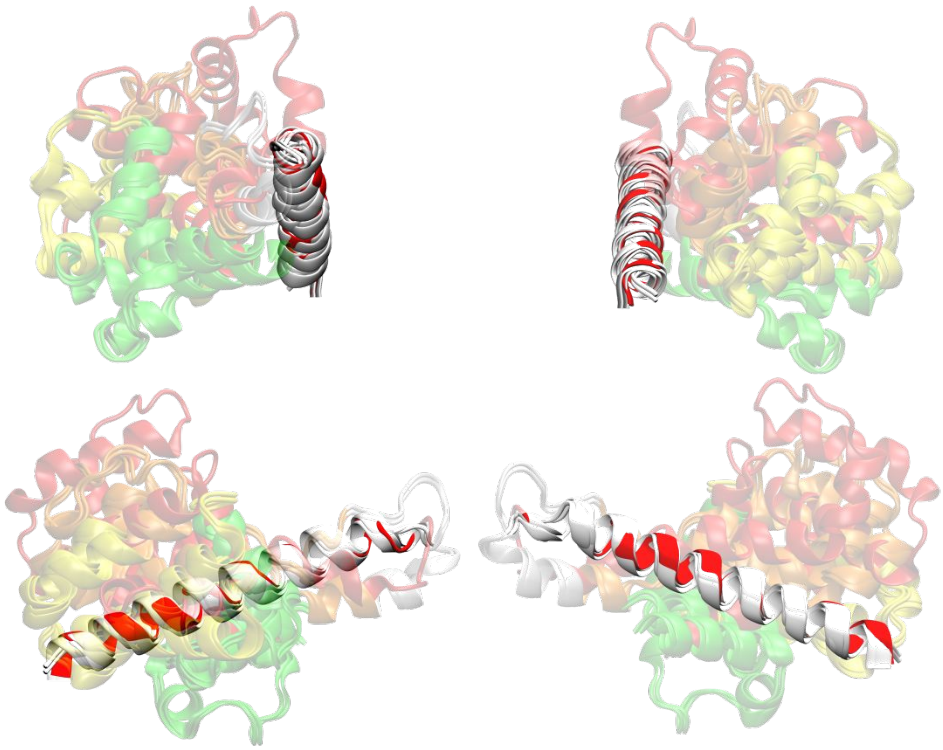

Figure S3. Structural alignment of residues 1–57 from Models 1–5 in different angles. Model 2 is highlighted in red for reference purposes. Residues 58–154 are shown transparent.

Table S1. RMSD values of Models 1–5 calculated for the entire protein, residues 1–57, and residues 58–154, relative to Model 2.

	**RMSD / Å**		
**Model**	**Protein**	**Residues 1–57**	**Residues 58–154**
**1**	2.79	1.06	0.74
**2**	0.00	0.00	0.00
**3**	1.63	1.13	0.68
**4**	2.49	1.67	0.70
**5**	2.24	0.94	0.89

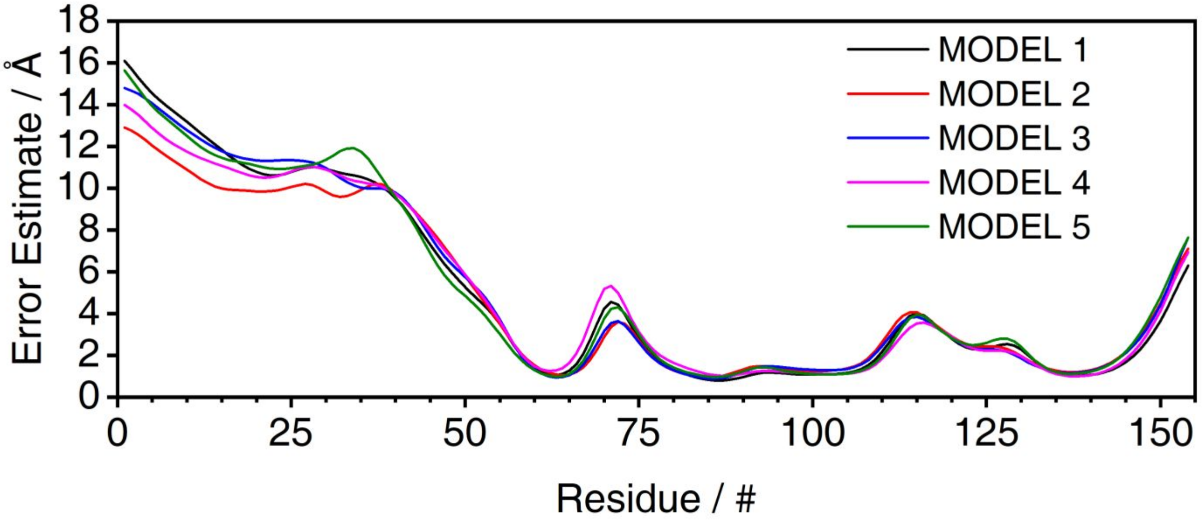

Figure S4. Residue Structural Error Estimate profile for the five generated models of P21.

	Model 1		Model 2		Model 3		Model 4		Model 5	
Residues in most favoured regions [A,B,L]	127	93.4%	131	96.3%	130	95.6%	130	95.6%	121	89.0%
Residues in additional allowed regions [a,b,l,p]	8	5.9%	4	2.9%	5	3.7%	3	2.2%	8	5.9%
Residues in generously allowed regions [~a,~b,~l,~p]	1	0.7%	1	0.7%	0	0.0%	0	0.0%	5	3.7%
Residues in disallowed regions	0	0.0%	0	0.0%	1	0.7%	3	2.2%	2	1.5%
	----	------	----	------	----	------	----	------	----	------
Number of non-glycine and non-proline residues	136	100.0%	136	100.0%	136	100.0%	136	100.0%	136	100.0%
Number of end-residues (excl. Gly and Pro)	2		2		2		2		2	
Number of glycine residues (shown as triangles)	10		10		10		10		10	
Number of proline residues	6		6		6		6		6	
	----		----		----		----		----	
Total number of residues	154		154		154		154		154	

Figure S5. Percentage distribution of residues for the five generated models of P21 in the Ramachandran plot.

Based on Figures S1–3 and Table S1, the models were highly similar, with the primary difference observed in the N-terminal region, as anticipated from the error estimates provided by the Robetta webserver (Figure S4). While some differences were observed, the similarity remained significantly high among the RoseTTAFold models. Variations were limited to the angulation in the helix structure formed by residues 1–57 (Figures S1). Due to the high similarity, Model 2 was selected for further simulations given it showed the lowest residue structural error estimate (Figure S4) and the highest percentage of residues in the most favored regions in the Ramachandran plot (Figure S6).

1.2. Comparison between RoseTTAFold and AlphaFold3.

Model 2 from RoseTTAFold and the model from AlphaFold3 are presented in Figure S6, with the former shown in red and the latter in blue.

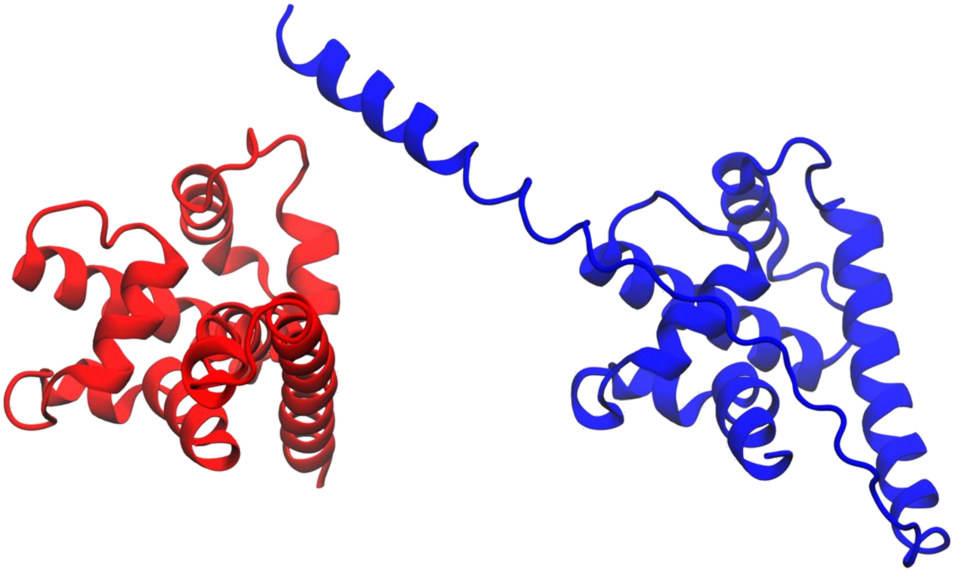

Figure S6. Models of the P21 protein as predicted by RoseTTAFold (shown in red) and AlphaFold3 (shown in blue).

After aligning both models, there is a clear conservation of residues 58–154 (representing 63% of the protein), as shown in Figure S7, with a RMSD value of 2.13 Å, leaving residues 1–57 open to interpretation.

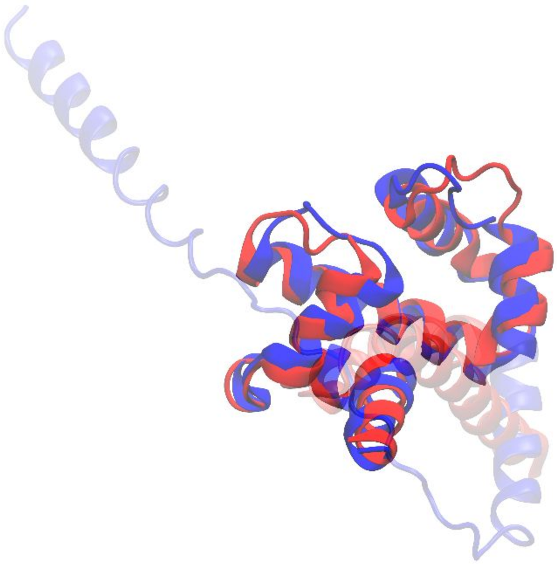

Figure S7. Highlighted alignment of residues 58–154 from the RoseTTAFold model (shown in red) and AlphaFold3 (shown in blue).

For residues 1–57, RoseTTAFold predicts a clear helical secondary structure, whereas AlphaFold3 indicates an overall irregular secondary structure. AlphaFold3, however, predicts a helical arrangement for residues 1–16, which gradually loses its organization, resulting in an irregular structure from 25–57. Notably, both RoseTTAFold and AlphaFold3 predict a similar arrangement up to residue 20, as shown in Figure S8, with an aligned RMSD of 2.10 Å.

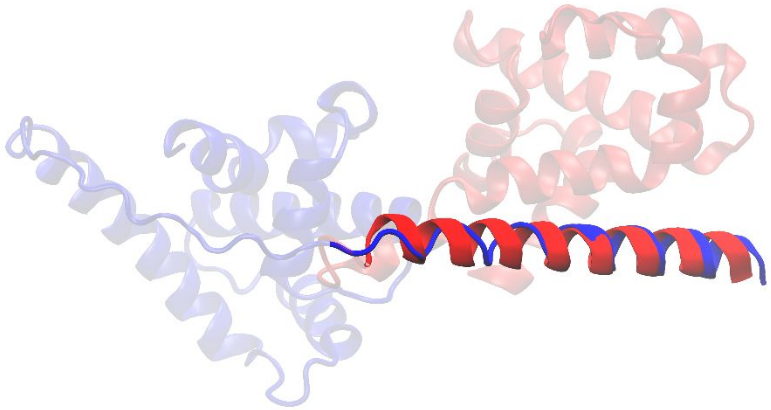

Figure S8. Highlighted alignment of residues 1–20 from the RoseTTAFold model (shown in red) and AlphaFold3 (shown in blue).

The computational tools employed for the structural quality assessment of the models predicted by RoseTTAFold and AlphaFold3 were Verify3D[1,2], ERRAT[3], and PROCHECK[4], all of which are available at the SAVES server. Each tool offers a different perspective for structural evaluation. Verify3D evaluates the correlation (3D-1D scores) between the 3D structure of the model and its corresponding 1D amino acid sequence; ERRAT provides an overall correlation profile for non-bonded interactions; and PROCHECK assesses the stereochemical quality of the model.

According to the Verify3D assessment (Figure S9), 80.52% of the RoseTTAFold model scored ≥0.1, in contrast to 59.74% of the AlphaFold3 model. RoseTTAFold exhibited 30 residues below the quality threshold, including residues 1–22, while AlphaFold3 showed 62 residues, including residues 1–61. Importantly, if evaluated solely using Verify3D, the AlphaFold3 model would fail the quality assessment.

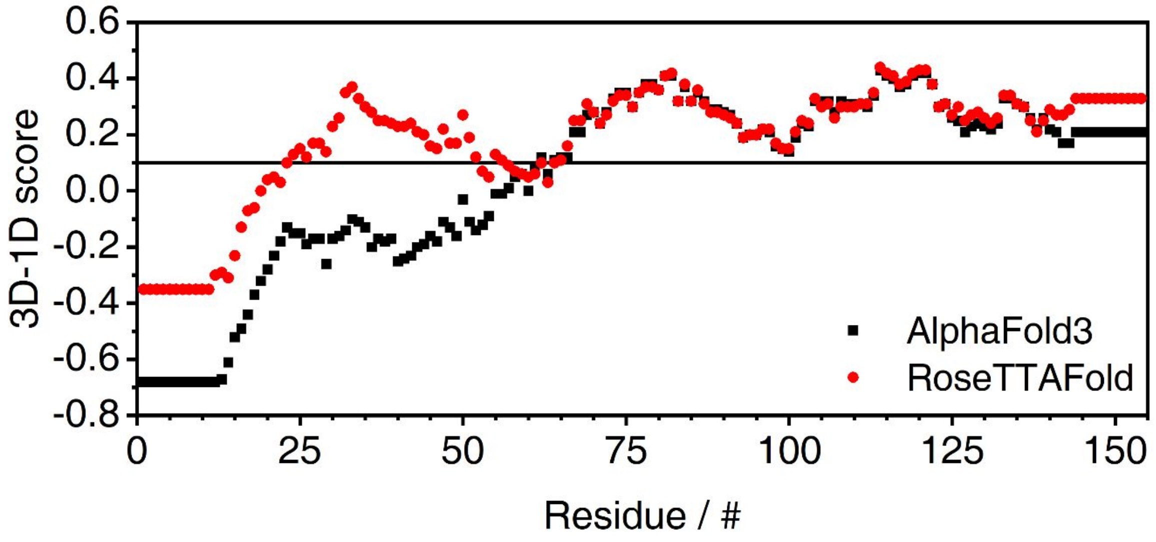

Figure S9. 3D-1D scores for each residue of the P21. The threshold line at 0.1 is indicated for clarity.

For the ERRAT assessment (Figure S10), the overall quality factors for the models from RoseTTAFold and AlphaFold3 were 100.0% and 71.0%, respectively. The residues are classified into four categories: (1) above the 99% confidence limit, (2) between the 95% and 99% confidence limits, (3) below the 95% confidence limit, and (4) below the minimum interaction limit. Residues in the first category are automatically rejected. Those in the second and fourth categories are questionable. Residues in the third category are considered acceptable.

After the software purposefully excluded the terminal residues (1–4 and 151–155), all 145 residues from the RoseTTAFold model were below the 95% confidence limit. In contrast, 103 residues from the AlphaFold3 model were below the same limit, while one residue (46) was between the 95% and 99% thresholds. Additionally, residues 44–45 were above the 99% confidence limit, and residues 5–43 were below the minimum interaction limit.

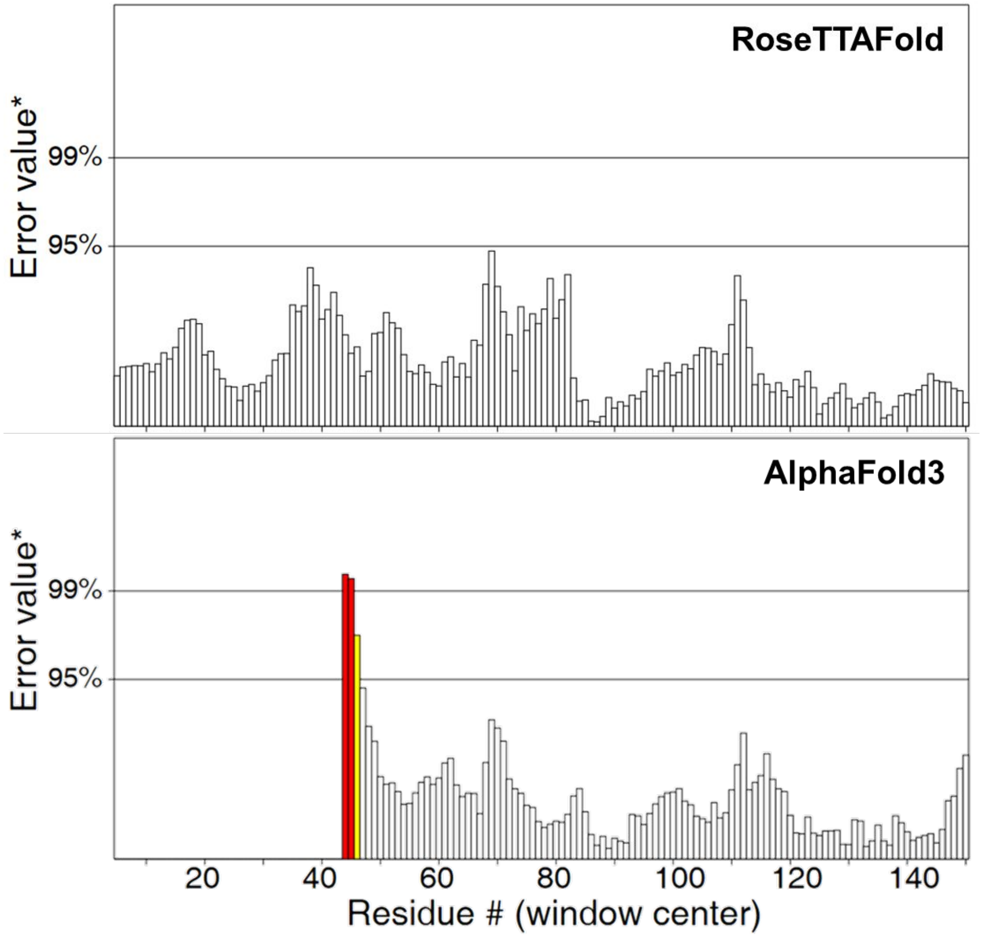

Figure S10. Overall quality factors from ERRAT for the analyzed models.

The results from the Ramachandran plot percentage distribution (Figure S11) were similar for both models. RoseTTAFold exhibited 131 residues (96.3%) in the most favored regions, while AlphaFold3 had 130 residues (95.6%).

	RoseTTAFold		AlphaFold3	
Residues in most favoured regions [A,B,L]	131	96.3%	130	95.6%
Residues in additional allowed regions [a,b,l,p]	4	2.9%	6	4.4%
Residues in generously allowed regions [~a,~b,~l,~p]	1	0.7%	0	0.0%
Residues in disallowed regions	0	0.0%	0	0.0%
	----	------	----	------
Number of non-glycine and non-proline residues	136	100.0%	136	100.0%
Number of end-residues (excl. Gly and Pro)	2		2	
Number of glycine residues (shown as triangles)	10		10	
Number of proline residues	6		6	
	----		----	
Total number of residues	154		154	

Figure S11. Percentage distribution of residues for the models from RoseTTAFold and AlphaFold3 in the Ramachandran plot.

When correlating the results, it becomes apparent that the major source of uncertainty in both models is in the N-terminal region. However, upon comparison, we considered that the model from RoseTTAFold presented less uncertainty. Given that the AlphaFold3 model did not pass two of the quality assessment tests and both algorithms have an outstanding credibility, the RoseTTAFold model was selected for further experiments.

2. SUPPLEMENTARY GRAPHICAL RESULTS

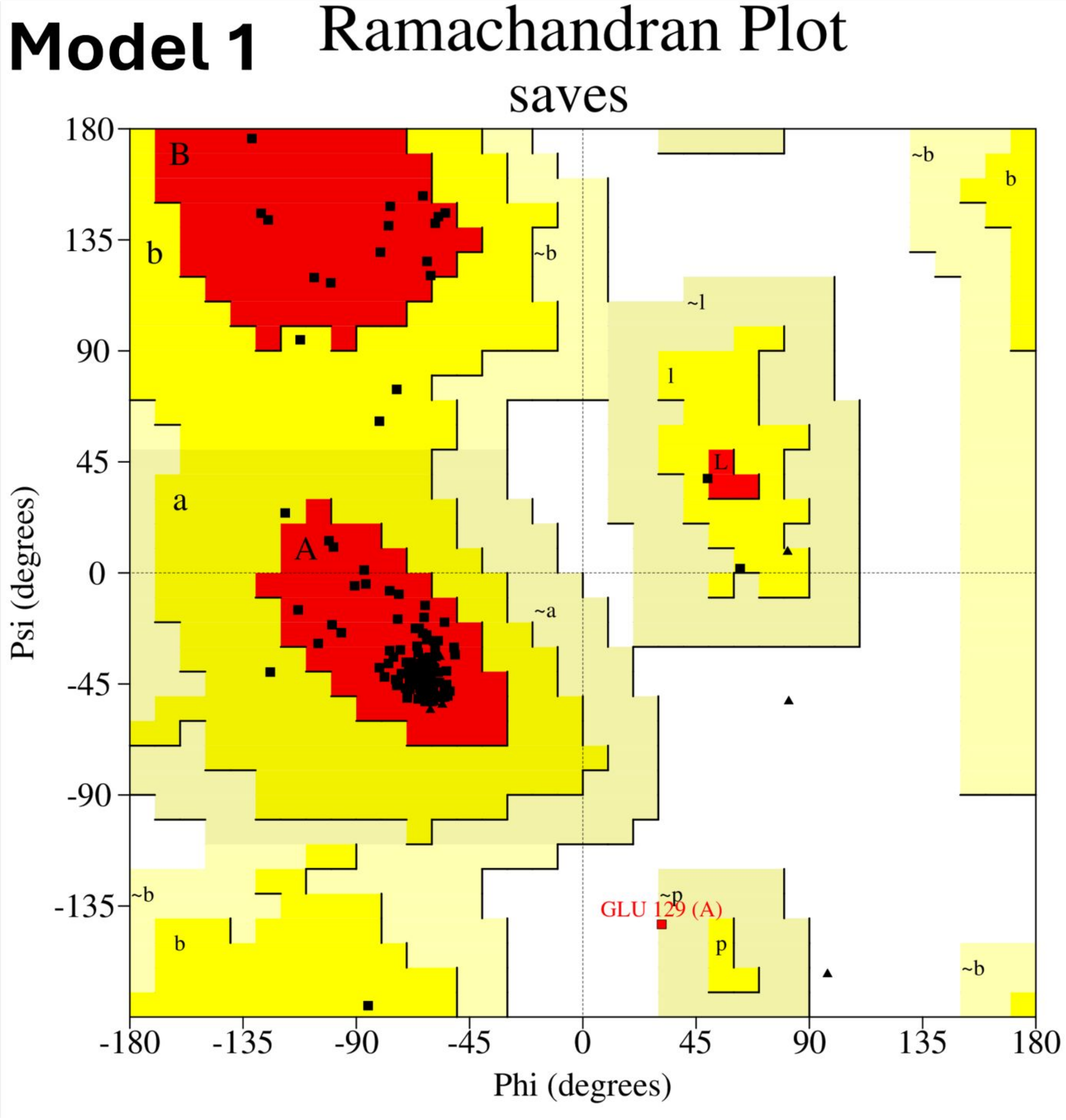

Figure S12. Ramachandran plot for P21 model 1.

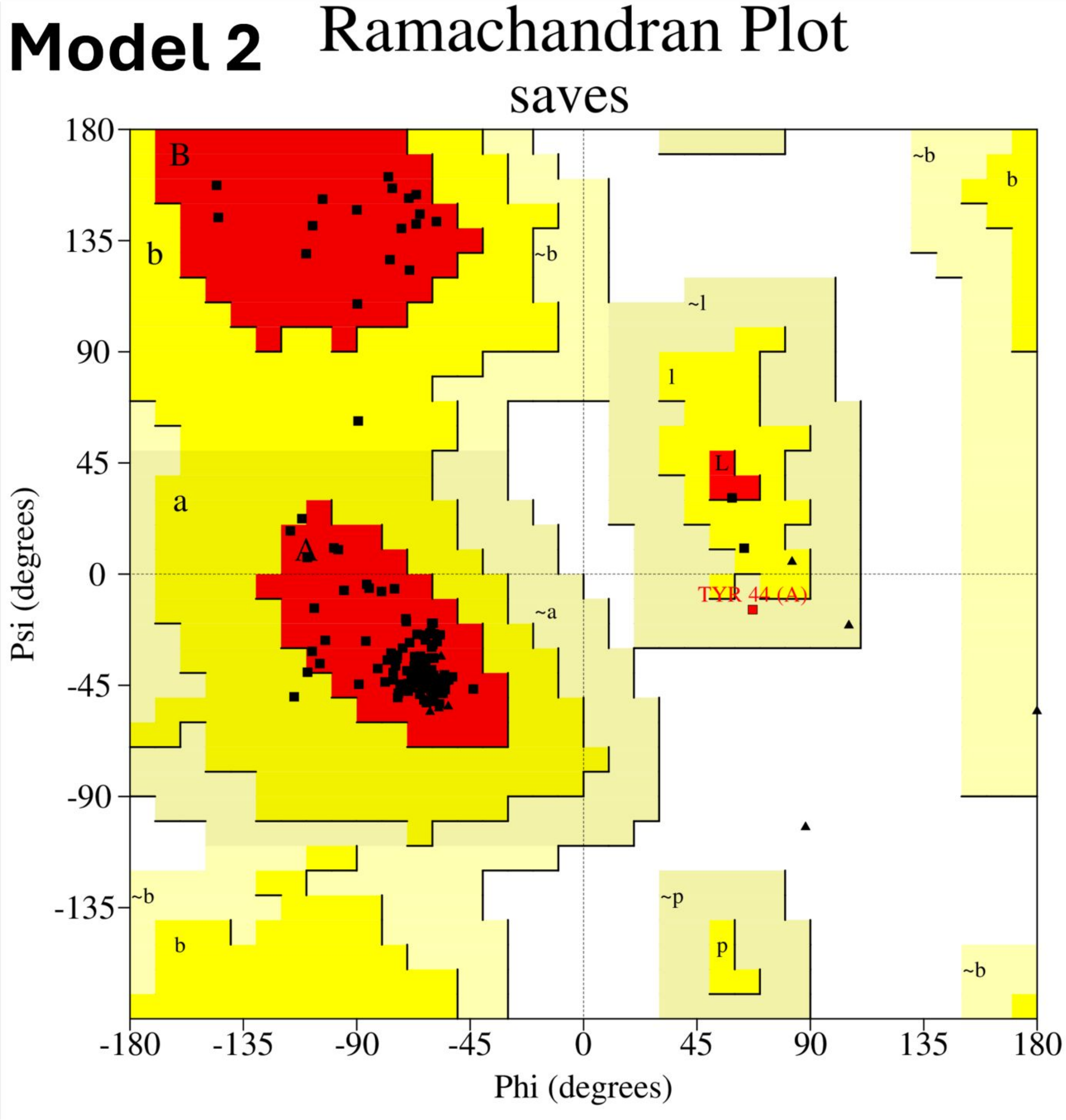

Figure S13. Ramachandran plot for P21 model 2.

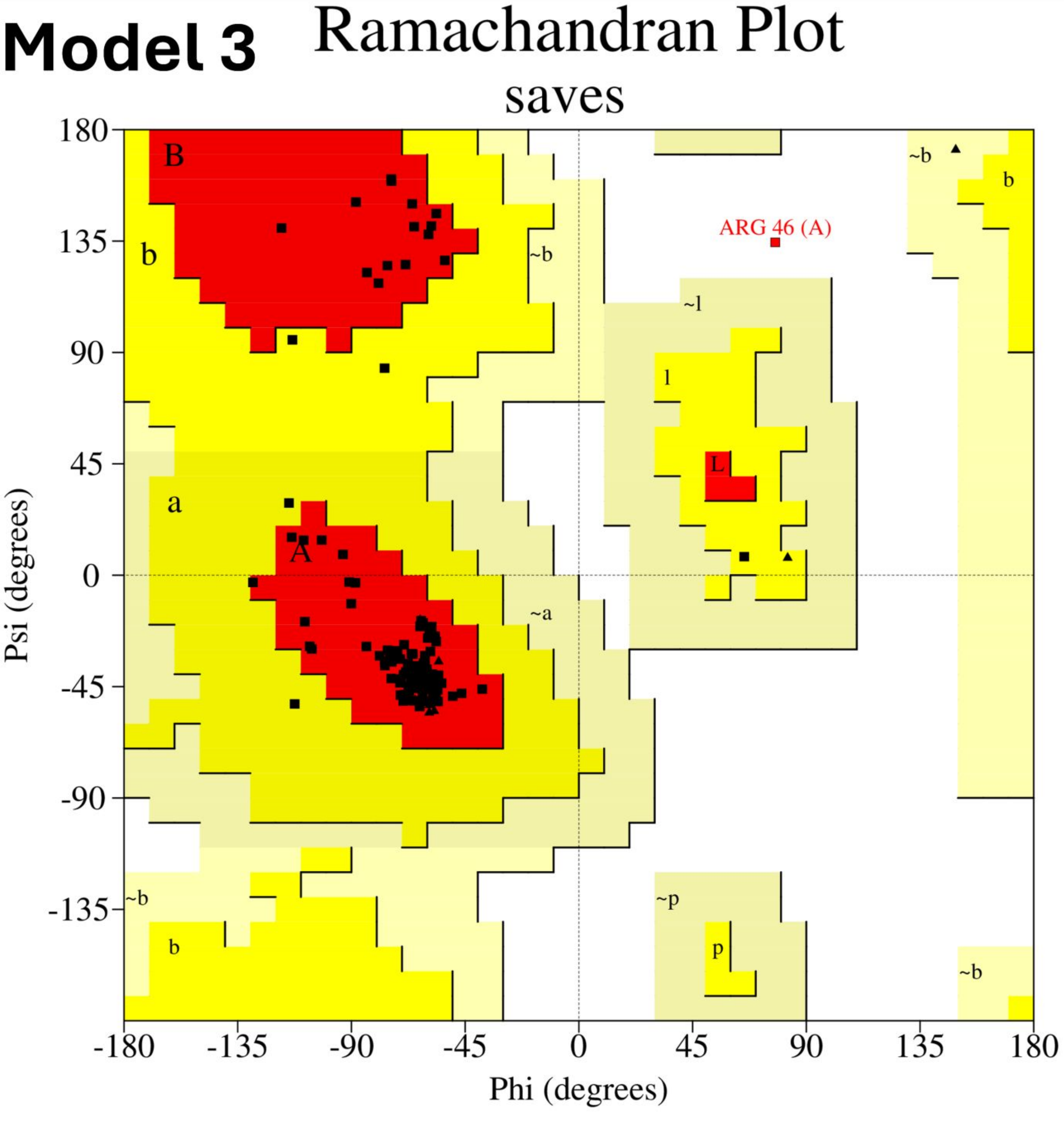

Figure S14. Ramachandran plot for P21 model 3.

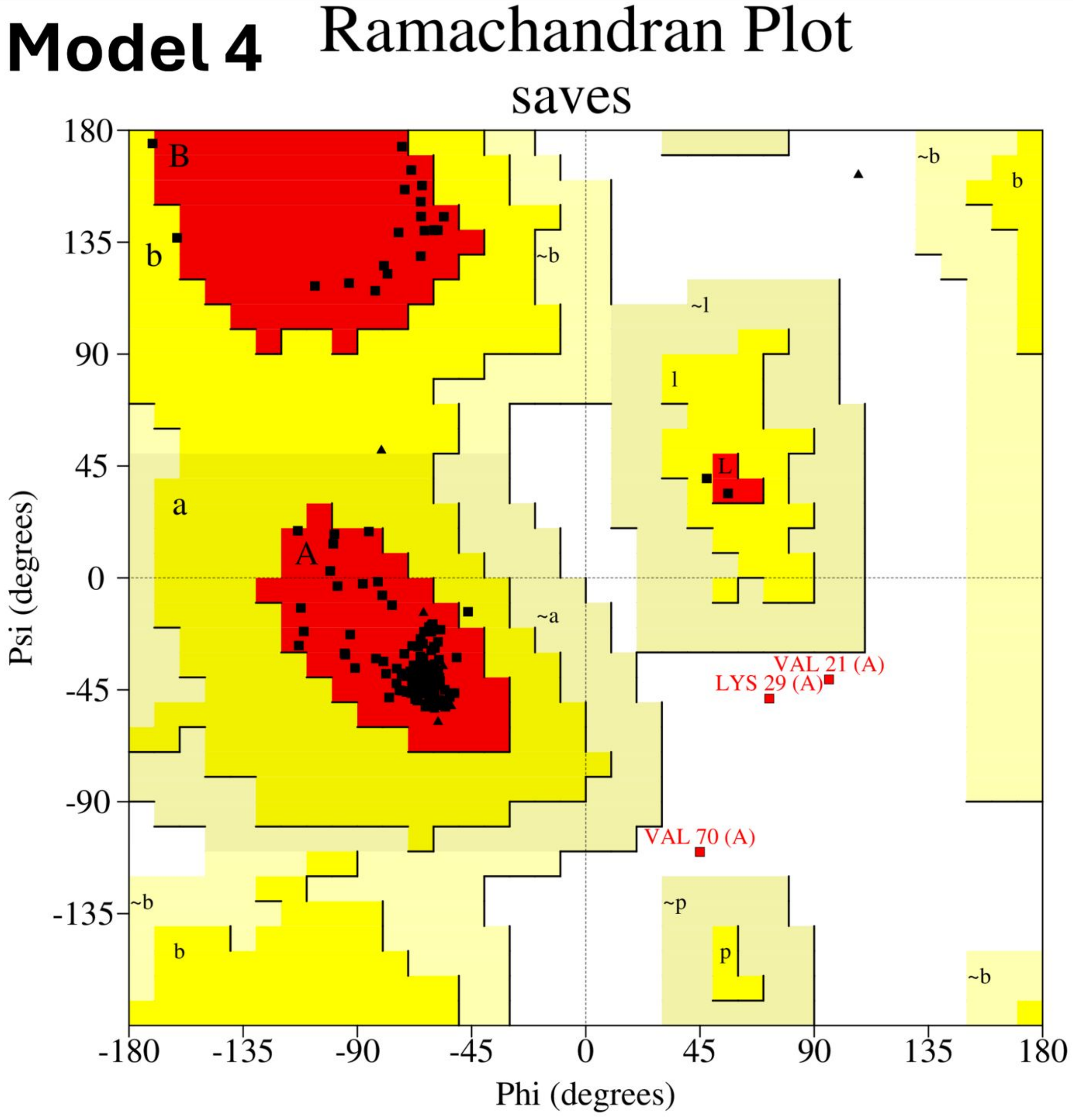

Figure S15. Ramachandran plot for P21 model 4.

Model 5

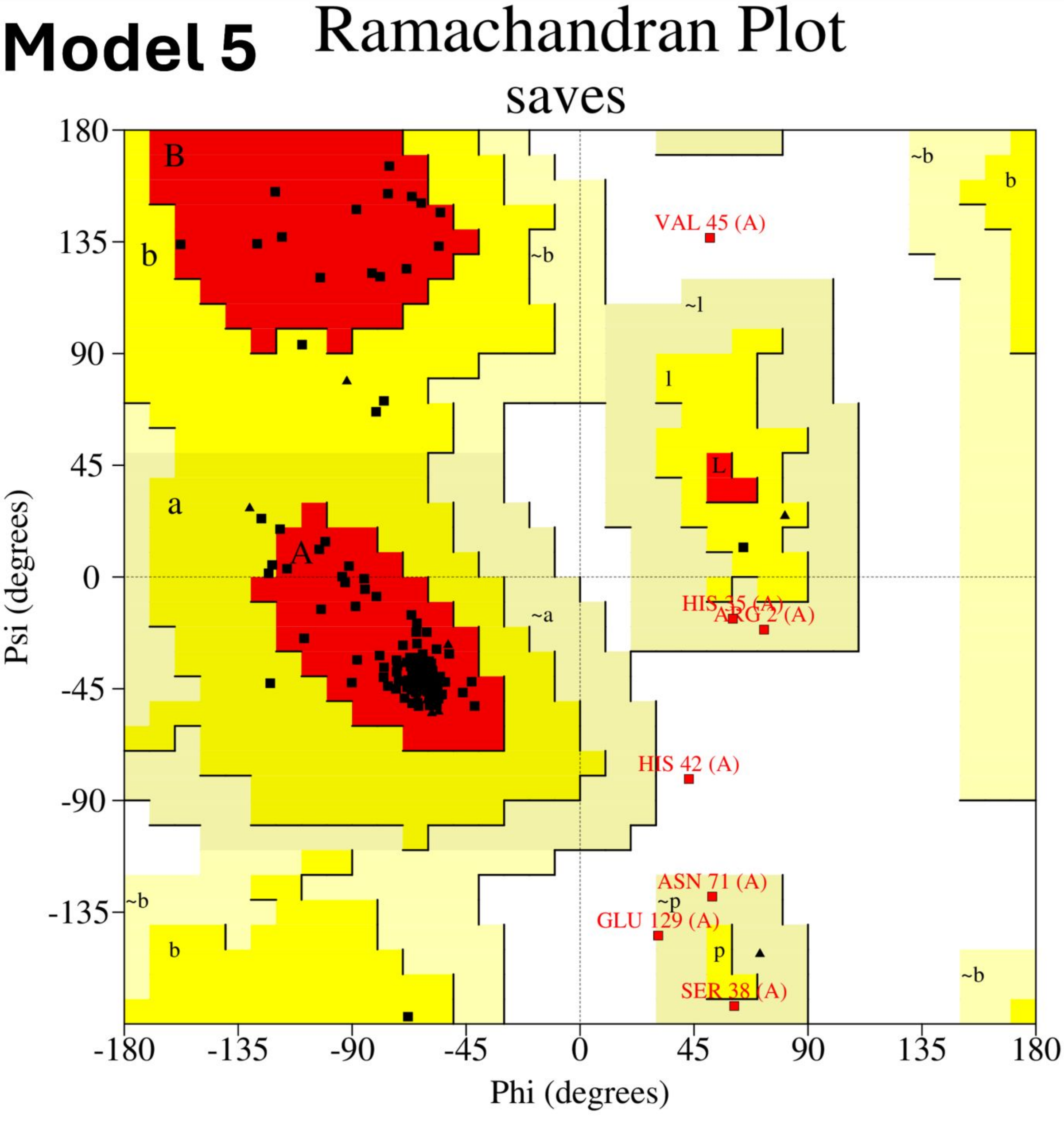

Figure S16. Ramachandran plot for P21 model 5.

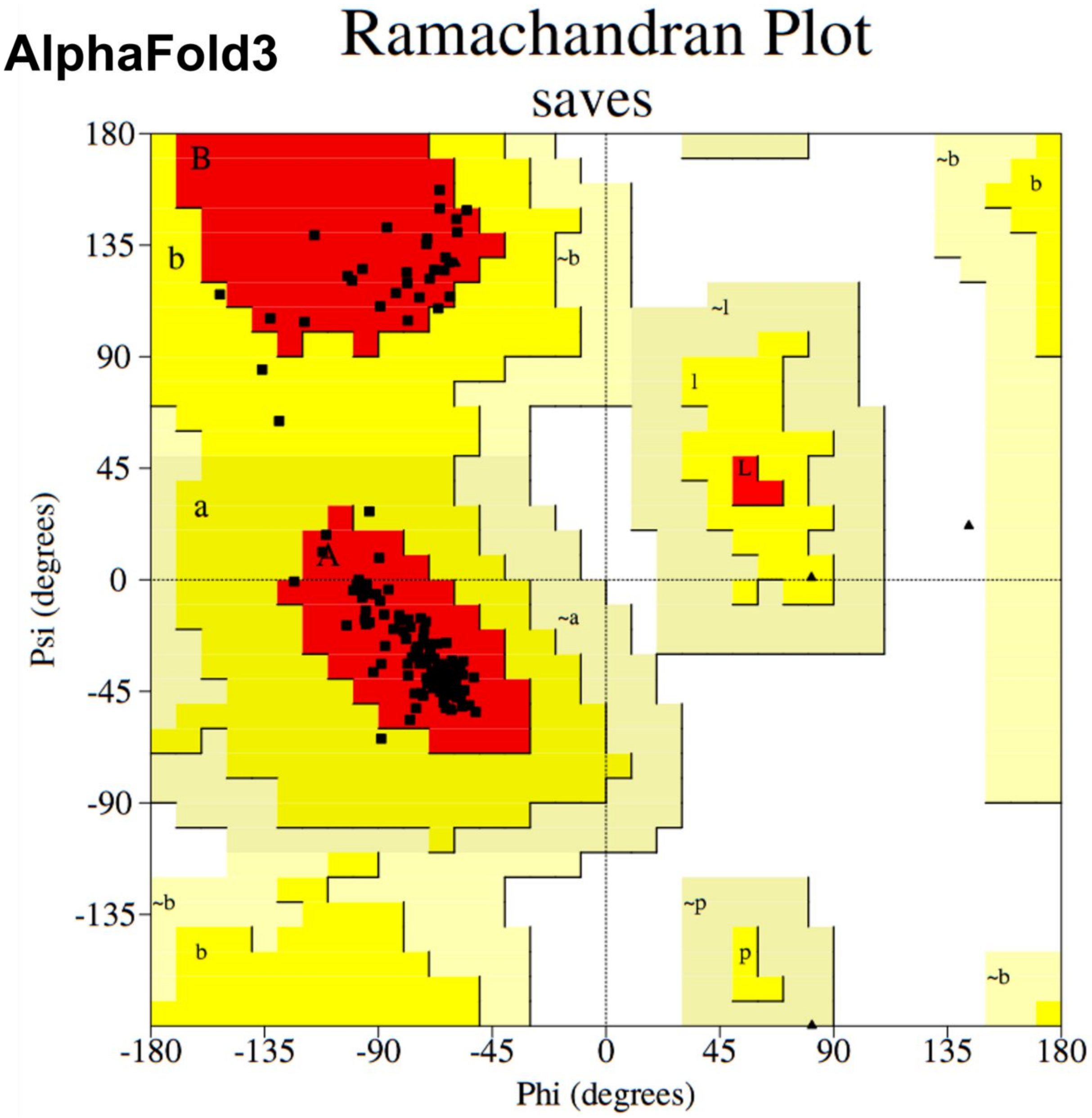

Figure S17. Ramachandran plot for P21 model from AlphaFold3.

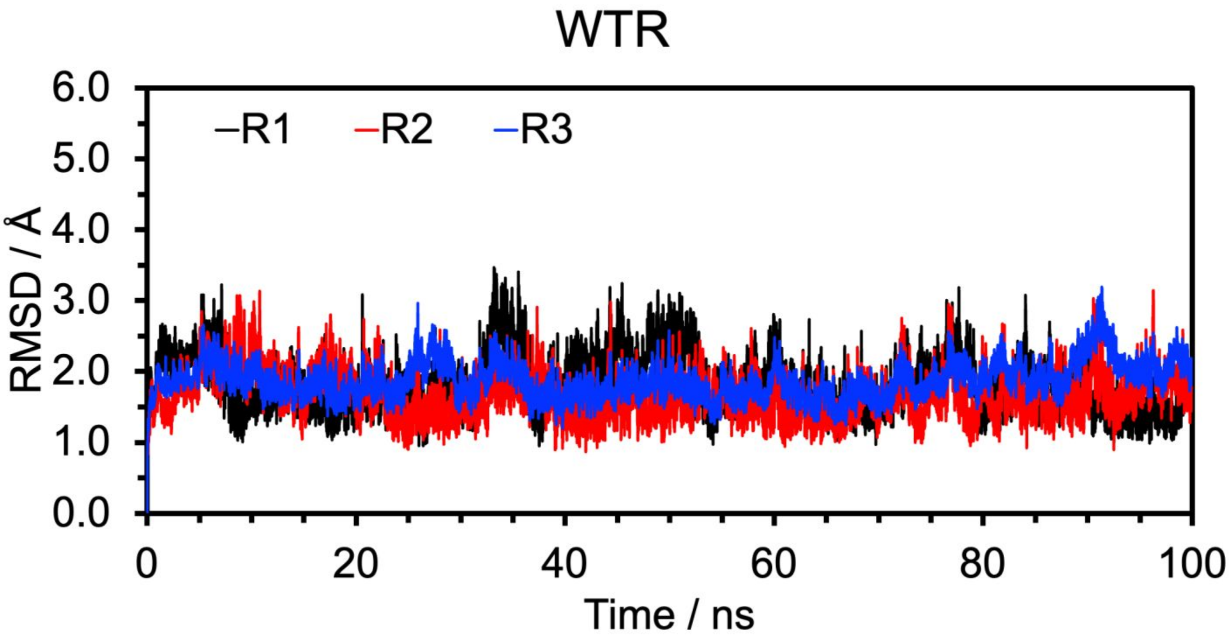

Figure S18. RMSD profiles for each replica of the P21 protein in water during equilibration.

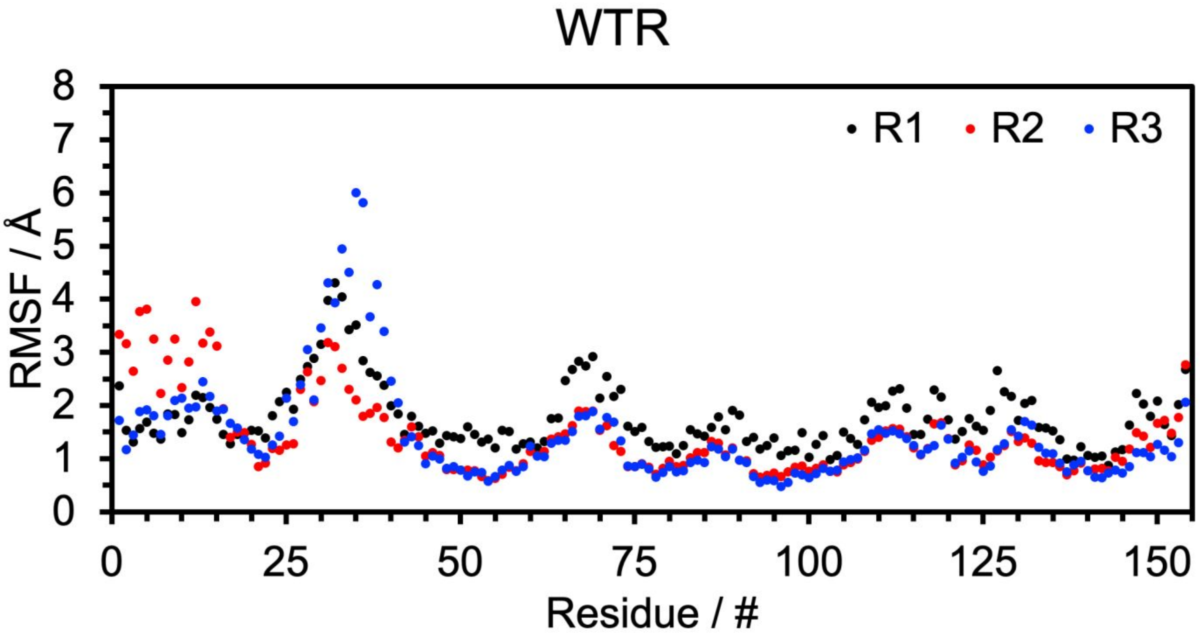

Figure S19. Per residue RMSF profile for each replica of the P21 protein in water during equilibration.

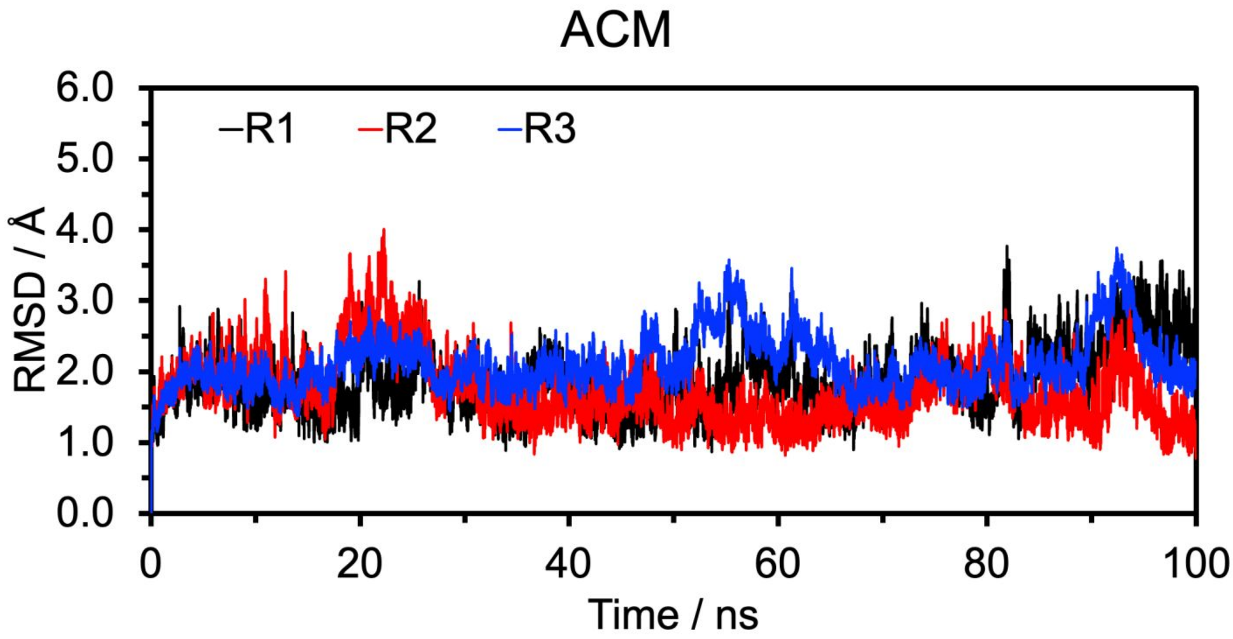

Figure S20. RMSD profiles for each replica of the P21 protein during the MSMD simulations with acetamide (ACM) as organic solvent.

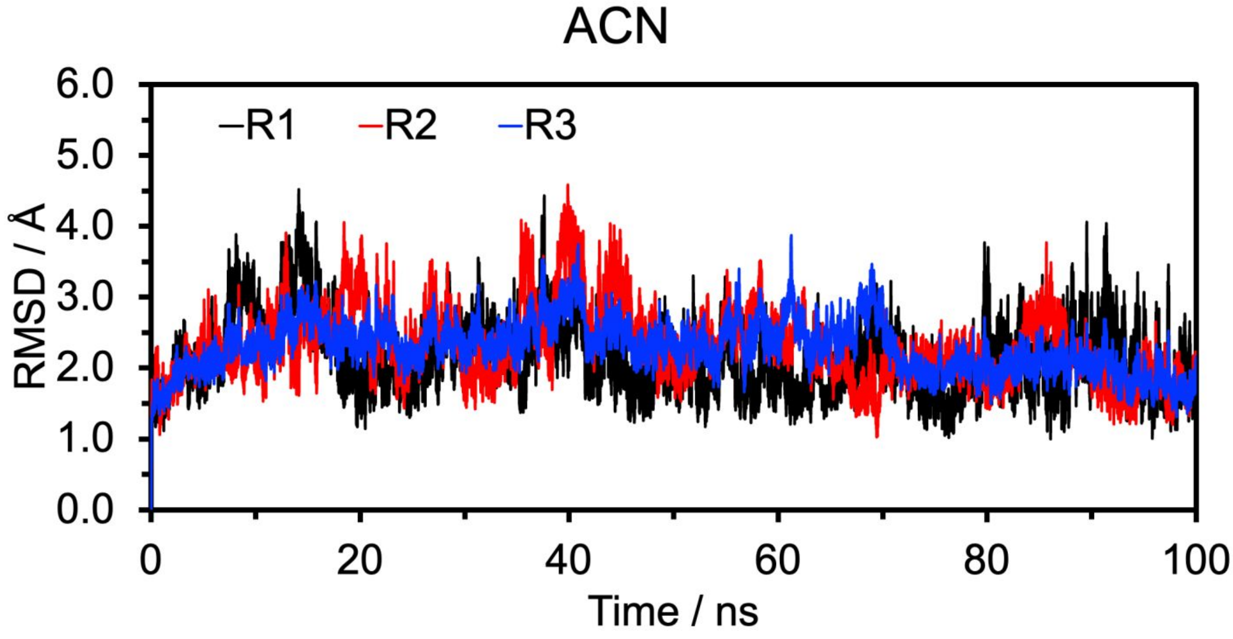

Figure S21. RMSD profiles for each replica of the P21 protein during the MSMD simulations with acetonitrile (ACN) as organic solvent.

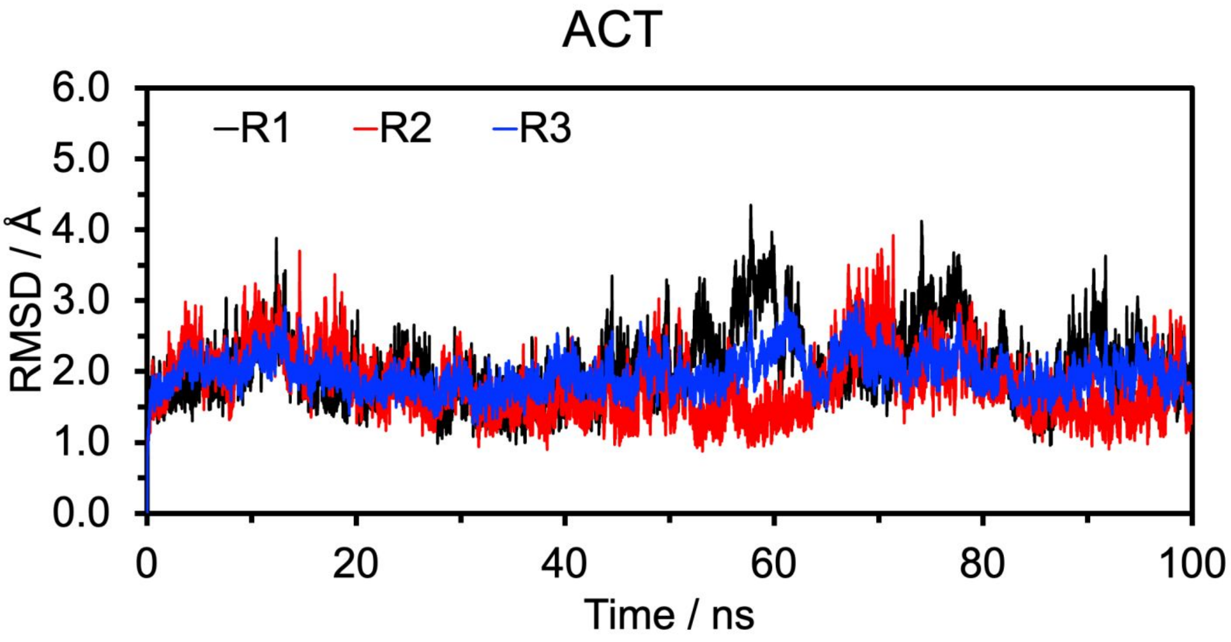

Figure S22. RMSD profiles for each replica of the P21 protein during the MSMD simulations with acetate ion (ACT) as organic solvent.

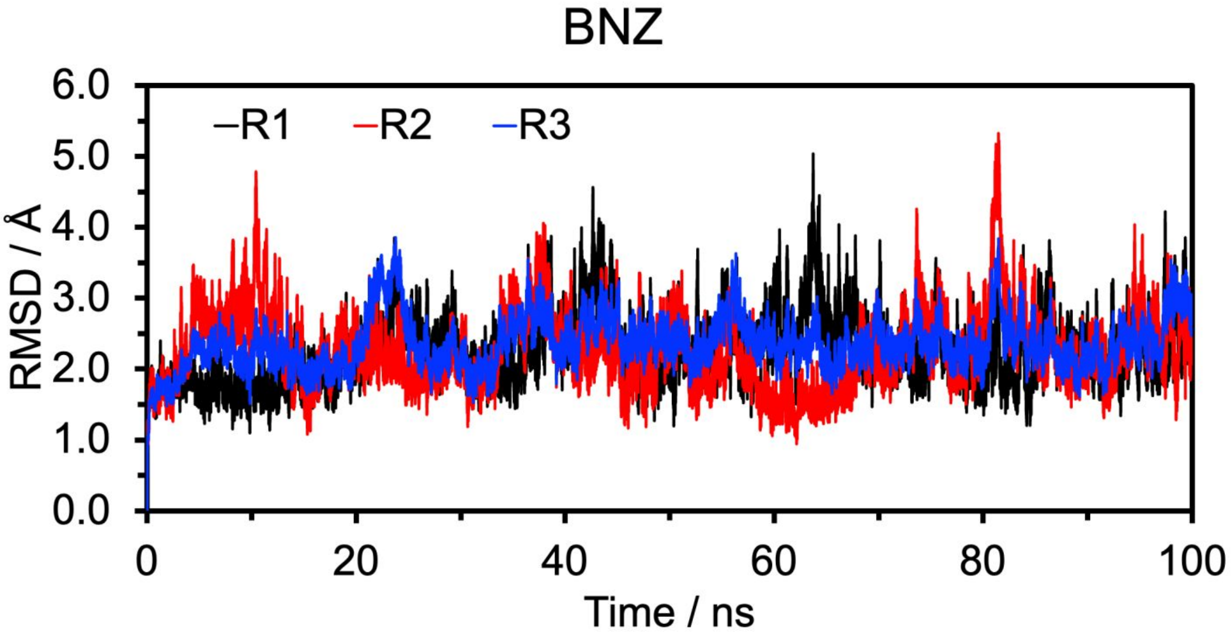

Figure S23. RMSD profiles for each replica of the P21 protein during the MSMD simulations with benzene (BNZ) as organic solvent.

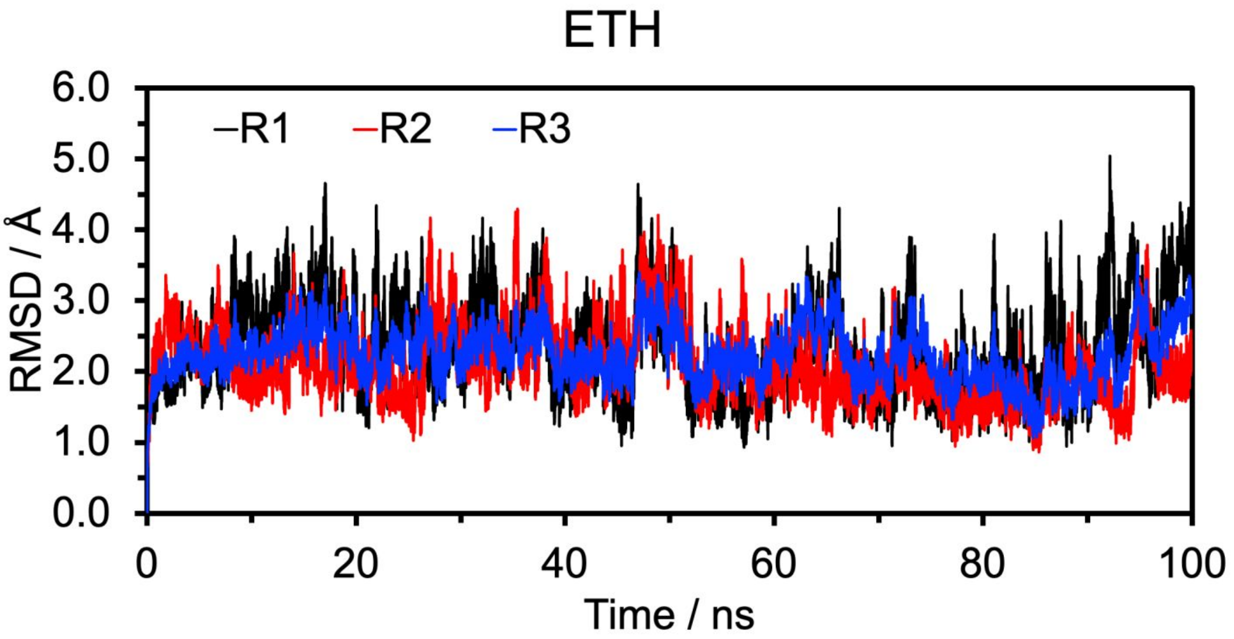

Figure S24. RMSD profiles for each replica of the P21 protein during the MSMD simulations with ethanol (ETH) as organic solvent.

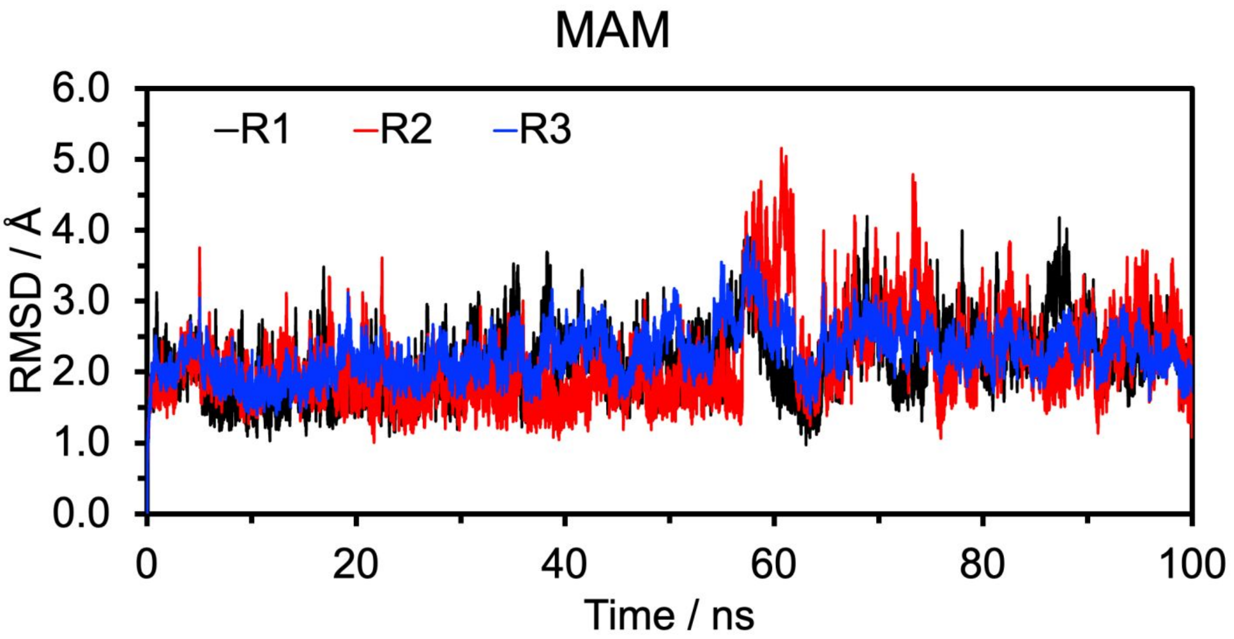

Figure S25. RMSD profiles for each replica of the P21 protein during the MSMD simulations with methylammonium ion (MAM) as organic solvent.

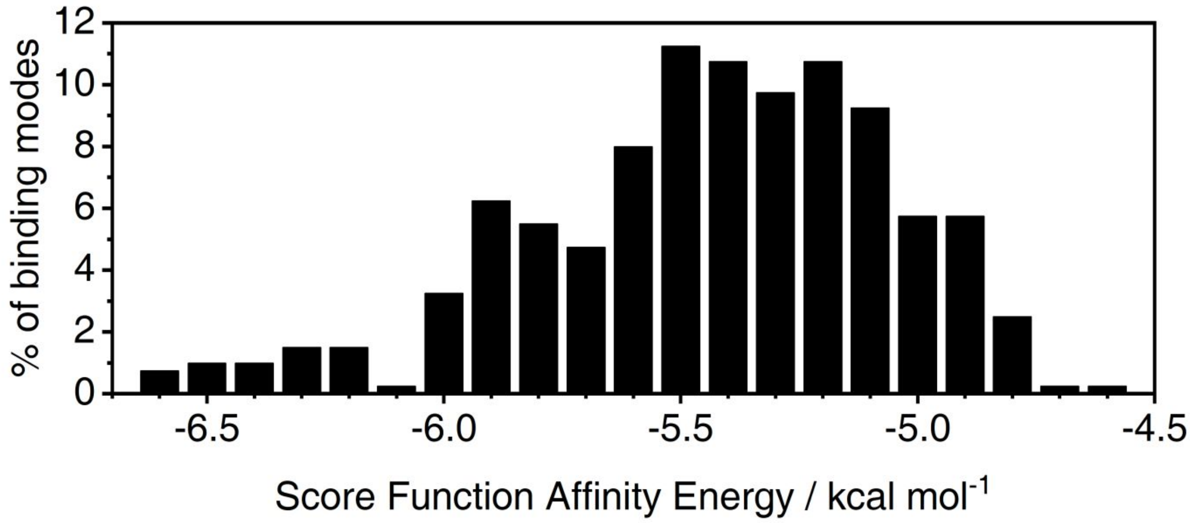

Figure S26. Score Function Affinity Energy Percentage Distribution.

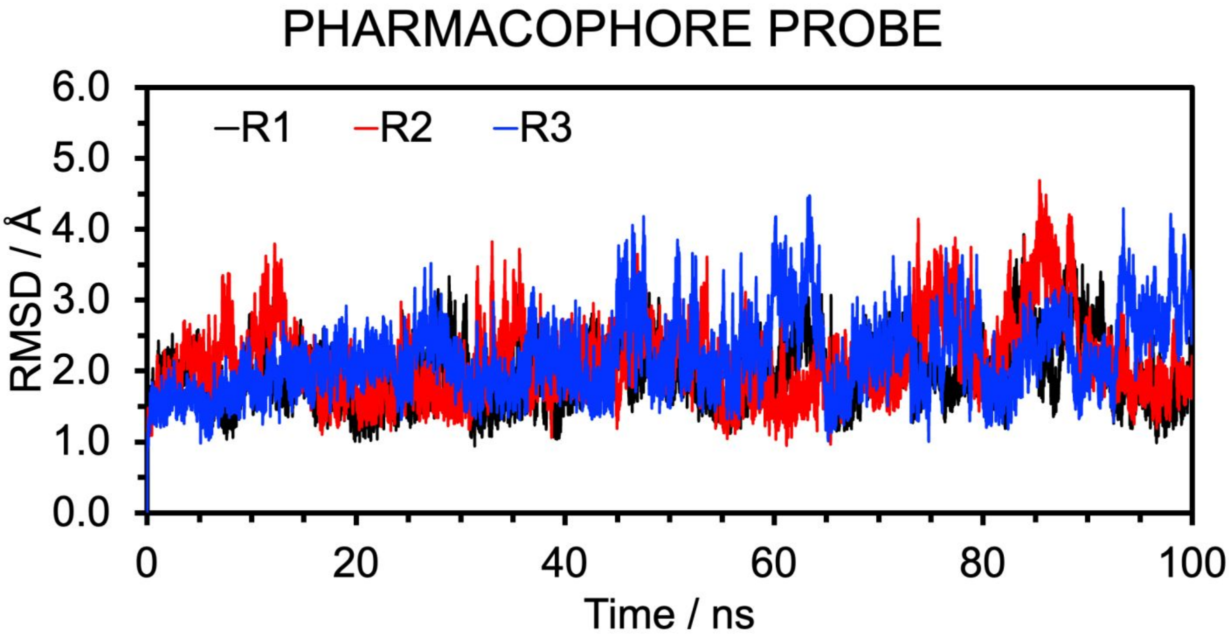

Figure S27. RMSD profiles for each replica of the P21 protein during the simulations with the pharmacophore probe model.

REFERENCES

(1) Bowie, J. U.; Lüthy, R.; Eisenberg, D. A Method to Identify Protein Sequences That Fold into a Known Three-Dimensional Structure. *Science* **1991**, *253* (5016), 164–170. https://doi.org/10.1126/SCIENCE.1853201.

(2) Lüthy, R.; Bowie, J. U.; Eisenberg, D. Assessment of Protein Models with Three-Dimensional Profiles. *Nature 1992 356:6364* **1992**, *356* (6364), 83–85. https://doi.org/10.1038/356083a0.

(3) Colovos, C.; Yeates, T. O. Verification of Protein Structures: Patterns of Nonbonded Atomic Interactions. *Protein Science* **1993**, *2* (9), 1511–1519. https://doi.org/10.1002/PRO.5560020916.

(4) Laskowski, R. A.; MacArthur, M. W.; Moss, D. S.; Thornton, J. M. PROCHECK: A Program to Check the Stereochemical Quality of Protein Structures. *J Appl Crystallogr* **1993**, *26* (2), 283–291. https://doi.org/10.1107/s0021889892009944.
